# Does Short-Term Hunger Increase Trust and Trustworthiness in a High Trust Society?

**DOI:** 10.3389/fpsyg.2017.01944

**Published:** 2017-11-07

**Authors:** Elias Rantapuska, Riitta Freese, Iiro P. Jääskeläinen, Kaisa Hytönen

**Affiliations:** ^1^Department of Finance, School of Business, Aalto University, Espoo, Finland; ^2^Department of Food and Environmental Sciences, University of Helsinki, Helsinki, Finland; ^3^Department of Neuroscience and Biomedical Engineering, School of Science, Aalto University, Espoo, Finland; ^4^Laurea University of Applied Sciences, Vantaa, Finland

**Keywords:** trust, reciprocity, trustworthiness, hunger, glucose, social heuristics hypothesis

## Abstract

We build on the social heuristics hypothesis, the literature on the glucose model of self-control, and recent challenges on these hypotheses to investigate whether individuals exhibit a change in degree of trust and reciprocation after consumption of a meal. We induce short-term manipulation of hunger followed by the trust game and a decision on whether to leave personal belongings in an unlocked and unsupervised room. Our results are inconclusive. While, we report hungry individuals trusting and reciprocating more than those who have just consumed a meal in a high trust society, we fail to reject the null with small number of observations (*N* = 101) and experimental sessions (*N* = 8). In addition, we find no evidence of short-term hunger having an impact on charitable giving or decisions in public good game.

## Introduction

Trust has been the grease in the wheels of historical and modern societies. Trust promotes economic growth, organizational efficiency, and innovation. The level of trust depends on beliefs about trustworthiness–individuals trust more if they believe their counterpart is trustworthy (Fehr, 2009). If individuals expect not to be cheated on and act accordingly, trading partners, acquaintances, or strangers can transact more smoothly with each other without the need of an explicit enforcement mechanism for every single contract.

The origins of trust can be investigated from several perspectives. One perspective is the environment an individual is exposed to. Environmental factors contributing to the choice of whether to trust include experiences (Nunn and Wantchekon, 2011; Stevenson and Wolfers, 2011; Peysakhovich and Rand, 2016), culture (Guiso et al., 2009; Bohnet et al., 2010), institutions (Algan and Cahuc, 2010; Alesina and Zhuravskaya, 2011), and even physical appearance (Duarte et al., 2012). The environment and individual lives in, and the clues on the trustworthiness of others she picks up, are important determinants of trust.

While determinants of trust can be attributed to the environment an individual is exposed to, biology provides another perspective. Trust and trustworthiness both have a genetic component (Cesarini et al., 2008; Riedl and Javor, 2012), they are affected by hormones (Kosfeld et al., 2005; Zak et al., 2005), and can be traced to distinct brain areas in functional magnetic resonance imaging (fMRI) representing fear of deception, anticipation of long-term benefits, mentalizing and rewarding experiences of being trusted (Rilling and Sanfey, 2011).

An important everyday biological process, hunger, has been until now virtually ignored in research for trust and reciprocity. However, empirical evidence on visceral factors mitigating or inhibiting trust is so far limited to physical temperature and sleep deprivation. Higher temperature has been associated with increased trust in others and prosociality in some studies (Williams and Bargh, 2008; Kang et al., 2011; although the results of the former study failed to replicate in Lynott et al., 2014). In contrast, after 36-h total sleep deprivation individual are less likely to place trust on others (Anderson and Dickinson, 2010; Dickinson and McElroy, 2017).

We contribute to this limited body of knowledge on the role of visceral factors in decision making by investigating how trust and trustworthiness are influenced by an everyday visceral factor, short-term hunger^1^. We concentrate on short-term hunger in this study since it is societally important: most individuals experience it every day. Furthermore, short-term hunger is generally not considered an issue that needs to be urgently addressed. To our knowledge, we are the first to empirically investigate whether short-term hunger influences trust and trustworthiness.

Trust is highly context-dependent and varies between countries, ethnicities, and institutions individuals are exposed to (Fehr, 2009). According to the social heuristics hypothesis (SHH, first version in Rand et al., 2012), beneficial social behaviors become internalized as default heuristics that impact and guide behavior reasonably automatically (Rand et al., 2014; Peysakhovich and Rand, 2016). Trusting in a high trust society can for instance be the intuitive behavioral response. In this paper, we particularly concentrate on short-term hunger in a high-trust society. Finland, where our data originate from, consistently ranks very high in interpersonal trust according to the World Values Survey placing third in the fifth wave of the survey.

To study whether short-term hunger influences trust and trustworthiness we observe choice behavior in a laboratory. Similar to recent work in social sciences (see Coleman, 1990; Fehr, 2009), we collect data on trust and trustworthiness behavior using the trust game (Berg et al., 1995) and by observing whether an individual leaves belongings in an unlocked and unsupervised laboratory room. In the laboratory, we compare decisions by sated healthy Finnish subjects after consuming breakfast with decisions by subjects consuming only water.

We offer two contributions to the literature. First, this study is the first that links short-term hunger with trusting and reciprocal behavior. While low blood glucose levels or short-term hunger have been previously linked in resource depletion models with decisions requiring self-control (Gailliot and Baumeister, 2007; Kuhn et al., 2017), risk taking (Symmonds et al., 2010; de Ridder et al., 2014), intertemporal choice (Wang and Dvorak, 2010), and political views (Petersen et al., 2014), scholars have not yet linked short-term hunger with individual trust and trustworthiness. Second, although we fail to reject the null hypothesis of no effect, the results would not be inconsistent with the claim that hunger leads to *more* trusting and strategic behavior. The literature so far generally associates low blood glucose levels or short-term hunger with heightened greed (e.g., Briers et al., 2006; Gailliot and Baumeister, 2007), but recent evidence (de Ridder et al., 2014) also indicates that hungry individuals may be more capable of making strategic decisions. This is consistent with the notion from the recent literature pointing out that decision environment may have unanticipated effect direction and mechanism than suggested by the resource depletion models (Kuhn et al., 2017).

This paper proceeds as follows. In the next section, we introduce hypotheses for trust and trustworthiness. Section “Short-Term Hunger, Trustworthiness, and Trust in the Laboratory” presents results from an empirical study and Section “Conclusion” concludes.

## Development of Hypotheses

Trusting and reciprocating by being trustworthy are intertwined and correlated with cooperative behavior in general, such as altruism and cooperation in the public good game (PGG; Peysakhovich et al., 2014). In an environment in which agents are trustworthy and are expected to reciprocate, trustworthiness promotes a cycle of mutual trust. However, trust and trustworthiness have important conceptual differences. Trusting is an act of the sender voluntarily placing resources at the disposal of the trustee without any legal commitment from the latter (some authors dub as “strategic cooperation”, see e.g., Rand, 2016). The act of trust is generally associated with an expectation that the act will pay off in terms of the investor’s goals (Fehr, 2009), although this notion has been challenged in the more recent literature (Espín et al., 2016). In a one-shot game, trusting behavior may be regarded as self-interested, strategic behavior if the opponent reciprocates with high enough of a probability of making the investment worthwhile (Bohnet and Zeckhauser, 2004). In contrast, returning a favor by being a trustworthy second mover in a one-shot trust game is prosocial behavior through an act of positive reciprocity and cannot be explained by purely selfish motives (Sanfey, 2007; dubbed as “pure cooperation” in Rand, 2016). As a result, we also use the terms trustworthiness and reciprocity interchangeably.

Drawing from previous literature, it is possible to posit two opposite predictions on the relationship between short-term hunger, trust, and trustworthiness. Based on recent controversies in the literature, it is also possible that short-term hunger has no impact on trust or trustworthiness, or that results may be highly context-dependent. We discuss all these possibilities below.

### Hunger Increases Trust and Trustworthiness (H_*A*1_)

Since deviation from an intuitive choice requires energy and behavioral control (Fairclough and Houston, 2004), we construct our first alternative hypothesis by considering trusting and trustworthiness as automated behavior. We argue that if an individual experiences short-term hunger, he or she would be more inclined to use less cognitive control and rely on the automated behaviors.

Some of the recent developments [described in Zaki and Mitchell (2013); also referred to as social heuristics hypothesis, or SHH, by Rand et al. (2013, 2014), Rand (2016), formalized in Bear and Rand (2016) and Bear et al. (2017)] on prosociality suggest that prosocial behaviors are automated and intuitive, although not always for all people. Individuals act more prosocially in experiments priming for the use of intuitive reasoning through time pressure and constrained ability to exert control. Bear and Rand (2016) as well as Bear et al. (2017) formalize this argument in their models by setting a cost, *d*, of exerting deliberation over intuition. Deliberation only pays off if the benefits of deliberation exceed a threshold *T*. Hence, as long as payoffs are small and costs of cognitive processing exceed potential gains, individuals cooperate intuitively if they come from an environment where cooperation is an equilibrium strategy. In addition, the argument by Rand (2016) would strengthen the expectation of observing increasing trust and trustworthiness in hungry individuals. Trusting involves future consequences and thus an impairment of cognitive processing such as induction of short-term hunger should increase trust and trustworthiness.

Preference for prosociality and fair sharing of resources may extend to the behavior of non-human primates (Brosnan and de Waal, 2003; former study contested in Roma et al., 2006) and the presence of other humans or human like features such as eyes accentuates prosociality in choices even in the absence of reciprocal social gratification (Burnham and Hare, 2007). These findings also corroborate the argument of prosocial and reciprocal behavior as intuitive, automated behavior. If short-term hunger induces individuals in the laboratory to resort to the automated behavior, they may be intuitively inclined to trust and reciprocate more than sated individuals who have more energy reserves to engage in controlled and effortful processes.

Recent developments in SHH (Rand, 2016) have made a distinction between strategic (such as trusting) and pure (such as reciprocating) cooperation. This line of thought argues that for strategic cooperation, inducing cognitive impairment would not have an impact as for pure cooperation. We would hence expect a stronger impact on trustworthiness than trust (if any) in the laboratory, because trusting induces no cognitive conflict if trusting is purely strategic behavior. However, there still may be a positive impact on trust, especially if relying on the early, more broad version of SHH (Rand et al., 2012) which does not incorporate cognitive conflict. In addition, trusting *may* be strategic, but it does no *have to* be strategic for all individuals.

### Hunger Decreases Trust and Trustworthiness (H_*A*2_)

It is possible to argue that hunger decreases trust and trustworthiness using three different lines of thought in our second alternative hypothesis. The first line of thought would use similar argumentation as above but with the opposite conclusion: not trusting or reciprocating is the default response and cognitive impairment such as short-term hunger would tilt toward less trusting and reciprocating behavior. The second line of thought draws from literature associating low blood glucose with heightened greed and impulsiveness. The third line of thought comes from evolutionary psychology.

Some scholars argue that prosocial behaviors are not automatic but trusting and reciprocating require cognitive effort. The proponents of the reflective model (e.g., Moore and Loewenstein, 2004; DeWall et al., 2008; Steinbeis et al., 2012; defined in Zaki and Mitchell, 2013) argue that humans act prosocially by suppressing the urge to act selfishly. This suppression requires energy and hence hungry individuals with reduced energy levels would be less likely to act prosocially. The expectation that not trusting is the default choice stems for instance from the literature on human development which suggests that not trusting others is the hard-wired standard response present in childhood. This tendency diminishes with experience: trusting behavior increases throughout childhood, and remains at a reasonably steady level in adulthood (Sutter and Kocher, 2007; van den Bos et al., 2010). Rand et al. (2014) make a related argument in laboratory: individuals with more laboratory experience cooperate less in one-shot experiments. This suggests that even if trusting and reciprocity are default choices at birth, individuals may update their priors based on experience and not trust and reciprocate as a default.

Low blood glucose levels have been associated with heightened greed, such as decreased self-control, impulse inhibition, and greater discounting of the future (e.g., Briers et al., 2006; Muraven et al., 2006; Gailliot et al., 2007; Hagger et al., 2010; Symmonds et al., 2010; Wang and Dvorak, 2010). Heightened greed and decreased self-control thus leave more room for selfish urges when individuals are hungry.

Some authors arrive at the same conclusion using argumentation from evolutionary psychology. Low blood glucose levels are linked to changes in behavior consistent with promoting strategies fit for survival. Increased risk-taking (Symmonds et al., 2010; Wang and Dvorak, 2010) and more impulsive behavior driven by lower self-control (e.g., Read and van Leeuwen, 1998; Gailliot and Baumeister, 2007) have been linked to depletion of energy resources in the body to promote more aggressive and risk-taking foraging strategies. Even the classic Maslow (1943) need hierarchy model would indicate that individuals subject to primary physiological needs such as hunger would prioritize immediate food acquisition (1st level of the hierarchy) to more future-oriented reciprocal behavior (2nd level in the need hierarchy) requiring self-control and risk-tolerance.

In conclusion, from these three arguments we could posit that increasing short-term hunger would lead to decreased trust and trustworthiness.

### Hunger Changes Trust and Trustworthiness Depending on Context (H_*A*3_)

Based on the discussion above, it is possible to posit trusting and trustworthiness either as automated or controlled process. This discrepancy could be explained by the fact that trust is highly context-dependent and varies between countries, ethnicities, and institutions individuals are exposed to (Fehr, 2009). Another argument leading to same conclusion leans on different types of social cooperation. A recent meta study by Rand (2016) argues that for pure cooperation, such as reciprocation in the trust game, deliberation reduces cooperation. Hence, cooperation with few future consequences (such as being the receiver in a one shot trust game) of one’s actions would be impaired by manipulation increasing the cost of cognitive processing.

Our experiments take place in Finland, which is a high-trust society. It consistently ranks very high in interpersonal trust according to the World Values Survey placing third in the fifth wave of the survey. Trusting and reciprocating with a stranger is the social norm in Finland similar to other Nordic countries (Brandt and Svendsen, 2010). According to the SHH (Rand et al., 2014; Peysakhovich and Rand, 2016), we would expect hungry individuals to trust and reciprocate with their counterparts more in the laboratory. This is because the society reinforce trusting behavior strongly so that trust and reciprocating have evolved to be intuitive choices and also a strategically sound decisions in our participant pool.

### Hunger Has No Impact on Trust and Trustworthiness (H_*Null*_)

It is also entirely possible that short-term hunger manipulation does not change trusting and reciprocal behavior. This is the case if (a) there is no generalizable baseline in prosocial behavior (individuals have no default in trusting and reciprocating behavior) which would be influenced by cognitive load manipulation (such as short-term hunger manipulation), (b) short-term hunger manipulation has no impact on trust and trustworthiness, or a combination of (a) and (b).

For (a), the jury is still out as the literature on SHH is currently under controversy. Tinghög et al. (2013) fail to replicate the highly influential original study on SHH by Rand et al. (2012) reporting individuals who reach decisions faster to be more cooperative and thus predisposed to cooperation. Verkoeijen and Bouwmeester (2014) report evidence inconsistent with the SHH. Furthermore, Bouwmeester et al. (2017) also fail to replicate the Rand et al. (2012) result in a multilab setting.

For (b), scholars have recently proposed that blood glucose levels may not have an impact on behavior. Resource depletion through lower blood glucose level may not lead to lower self-control and thus more likely default response. Vadillo et al. (2016) challenge earlier studies linking glucose and self-control and present evidence on publication bias in this stream of literature. Lange and Eggert (2014) do not find evidence on lower glucose having a detrimental impact on self-control. Furthermore, Kurzban (2010) dissects the theoretical argumentation of the glucose model concluding that empirical evidence consistent with glucose not being a resource to willpower, but may be an input to the decision-making process. Some studies using cognitive load manipulation other than glucose report findings consistent with the idea that prosocial behavior is not influenced by cognitive load (Kessler and Meier, 2014; Mieth et al., 2016). On a higher conceptual level, these findings together with failed replications (Hagger et al., 2016) challenge the ego depletion model of self-control (Baumeister et al., 1998; Muraven et al., 1998).

In sum, the recently accumulated evidence on (a) and (b) cast doubt on the social heuristics hypothesis, at least in its original form (Rand et al., 2012) and the glucose model of self- control. Hence, based on the previous literature, it is also entirely possible that cognitive load manipulation through short-term hunger does not have an impact on trust and trustworthiness.

## Short-Term Hunger, Trustworthiness, and Trust in the Laboratory

### Short-Term Hunger, Trustworthiness, and Trust in Trust Game

#### Method

We recruited 101 participants (or *subjects*) from the University of Turku Public Choice Research Centre (PCRC) participant pool to participate in the study. We had a between-subjects single-blind incentivized (10 EUR show up plus up to 10 EUR performance fee) experimental design using four randomly assigned groups on four days with treatment (“sated condition”) consuming a meal and four groups on four days with control condition (“hungry condition”) consuming only water before the experiment.

We did not have any reliable guidance on previous literature on expected effect sizes. Furthermore, the experimental setup was also rather invasive (adherence to an overnight fast and two blood glucose measurements). As a result, we recruited all participants we could from the participant pool, did not perform *ex ante* power calculations, and as a result any statistically insignificant results may be uninformative about the true effect. We outline the experimental procedure below and complete details of the laboratory experiment are given in Appendices A–C.

We requested all participants to adhere to an overnight fast in our call for participation and reminder sent 24 h before the laboratory session. Participants were informed about serving of a meal on the call for participation and the reminder, but they did not know the exact timing of the meal.

After arriving at the laboratory at 09:30 AM in each session, all participants completed pre-treatment measurements. First, they rated their hunger and satiety-related sensations (hunger, fullness, satiety, desire to eat, and prospective consumption) and thirst using 10 cm visual analog scales (VAS, see Blundell et al., 2010 and Appendix B1). Then, for verifying adherence to 10-h fast, an experienced nurse measured capillary blood glucose concentrations after which the participants were served either a meal (treatment group) or water (control group).

As our experimental manipulation intends to capture the effect of an experienced short-term hunger rather than a peak in glucose concentration, we decided to use a balanced meal to make the setting a more natural breakfast *versus* a skipped breakfast comparison. The meal consisted of a cheese sandwich, yogurt, and orange juice with an average energy content of 521 kcal (2190 kJ) per serving.^2^ Participants in the treatment condition were offered a meal before any post-treatment measurements while participants in the control condition were offered initially only water and a meal after all post-treatment measurements were completed.

We randomized at the session level and thus all participants in the same session were under the same experimental condition. When using conservative baseline metrics in the Section “Results and Discussion”, we assume observations are clustered within session. We also report results when relaxing this assumption.

After consuming a meal or water, the participants completed a survey with a questionnaire related to another study, a control questionnaire on emotional valence, and a second VAS-assessment at the end of the survey (Appendix B2). The nurse measured blood glucose concentrations for the second time after approximately 10 min after finishing the meal or drinking water. Next, the experimenter asked participants to proceed to the second laboratory room and casually mentioned “you can leave your belongings such as jackets and bags here or take them with you, it is entirely up to you.” This is a hidden experiment for an additional investigation of trust using the same participants.

We included the trust game with a multiplier of 3 and direct response method (see Appendix A1 and Berg et al., 1995 for details) as a part of the laboratory session in the second laboratory room (screenshots are given in Appendix C). The individuals completed the trust game first, followed by a prisoner’s dilemma (PD), a public goods game, and two dictator games with charitable giving to external recipients (for summary descriptions of all games, see Levitt and List, 2007). We used additional games to avoid replicating the rather expensive and somewhat invasive laboratory session in case requested by the scientific community. We did not hypothesize *ex ante* on these games, but decided to include them to not have to redo rather time-consuming, invasive, and expensive laboratory sessions in case requested by the research community. Our reasoning proved to have some merit ex post, as in fall 2015 we calculated an aggregate metric for pure cooperation to be included as a part of meta-study by Rand (2016)^3^.

Subjects completed three rounds of each game. We used experimental currency units (ECUs) in the experiment with ten ECUs corresponding to 1 EUR and the experiment was programmed and conducted with the experiment software z-Tree (Fischbacher, 2007).

Similar to Schotter et al. (1994), we were concerned about subjects not perceiving the trust game as a strictly one-off problem and either learning or establishing a norm of future play through their own actions. To alleviate such concerns, we randomized the counterparts and roles (with replacement) in each round to induce participants to consider each round and decision strictly as a one-off decision and limited the number of standard trust game rounds to three with a randomly chosen counterpart and role (sender or trustee) redrawn with replacement each round. Each participant would thus play a randomly assigned number (0–3) of rounds of the trust game as a sender and the remaining rounds as a receiver in the trust game.

In PD game, players have to decide whether to cooperate or defect. Players will achieve highest combined payoff (3 ECUs to both) by cooperating, but a player has always an incentive to cheat for a higher payoff. In case the other player cooperates, the defector gets 5 ECUs and the cooperator gets nothing. In case both players defect, the payoffs are 1 ECU to both players.

In PGG, both players receive 4 ECUs which they can partially or wholly contribute to common pool, which is increased by 50% by the experimenter and equally shared between participants. We use pairwise iteration so players will learn the outcome of the other player at the end of each round. Similar to PD, both players have an incentive to defect by not contributing to common pool.

Same as for the trust game, we randomized counterpart at the beginning for PD and PGG at each round and each game to induce strictly one-shot decision frames. The participants were also informed about the randomization in the verbal and written briefing (Appendix A3) prior to the experiment.

Two dictator games were used with charitable giving framing. The participants were allocated additional 5 ECUs that they could donate wholly or partially to the New Children’s Hospital 2017 project and a further 5 ECUs to be potentially donated to the Finnish Red Cross Disaster Relief Fund. After the two dictator games, new round would begin or at the end of third round the participant would be forwarded to a short survey including demographic questions (year of birth and gender), height, weight, and a question on leaving personal belongings in the first laboratory room. All participants were told after the survey that the experiment had been finished now and that they would be paid in an adjacent room. Finally, participants in the control condition were offered a meal after the survey.

#### Results for the Trust Game

Out of 101 participants, 60 are students and 76 females^4^. **Table 1** shows that gender, birth year, student indicator, and BMI are very similar across conditions implying participants did not incidentally cluster across demographics or physical qualities. They are on average 26 years old (born in 1988), and have an average BMI of 23.5 kg/m^2^. Furthermore, **Table 1** also shows that sated and hungry conditions have almost identical pre-treatment blood glucose concentration levels (5.02 mmol/L in hungry versus 5.01 mmol/L for sated condition, Eskelinen (2012) reports a reference level range of 4.0–6.0 mmol/L). The post-treatment blood glucose concentrations were slightly lower among the control condition subjects who were offered only water (4.84 mmol/L, not statistically significantly different from pre-treatment value) whereas the treatment condition subjects receiving food showed an increase to 6.78 mmol/L, which is a typical post-digestion level verifying that the meals were consumed. The difference in post-treatment glucose level between treatment and control conditions is statistically significant with *p* < 0.001 in a two-tailed *t*-test of means.

**Table 1:** Descriptive statistics for treatment (sated) and control (hungry) conditions.

		Min	Mean	Median	Max	*SD*	*N*
Indicator: male	Hungry	0.00	0.24	0.00	1.00	0.43	49
	Sated	0.00	0.25	0.00	1.00	0.44	52
Year of birth	Hungry	1972	1987	1988	1994	4.59	49
	Sated	1974	1988	1989	1994	4.36	52
Indicator: student	Hungry	0.00	0.57	1.00	1.00	0.50	49
	Sated	0.00	0.62	1.00	1.00	0.49	52
BMI (kg/m^2^)	Hungry	17.37	23.39	22.76	39.18	3.97	49
	Sated	16.73	23.53	22.50	46.25	4.54	52
Pre-treatment glucose (mmol/L)	Hungry	4.00	5.02	5.00	6.20	0.55	49
	Sated	3.90	5.01	5.00	7.20	0.62	52
Post-treatment glucose (mmol/L)	Hungry	3.50	4.84	4.80	6.70	0.75	49
	Sated	4.70	6.78	6.70	9.40	1.16	52
Group size	Hungry	8.00	14.37	14	20.00	5.11	49
	Sated	6.00	15.85	18	20.00	5.43	52


*This table reports descriptive statistics for individuals in the control (hungry) and treatment (sated) condition. Body mass index (BMI) is based on self-reported weight and height.*

**FIGURE 1:**
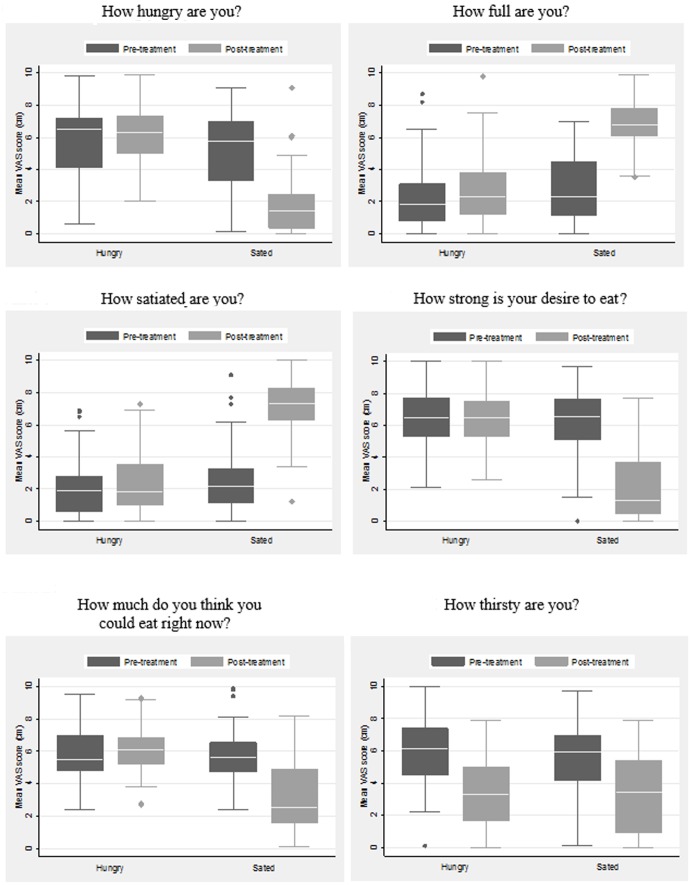
Box plots from Visual Analog Scale questions for control (hungry) and treatment (sated) conditions. The box indicates middle quartiles and white line denotes median. Bar widths show the range and dots show outlying observations. *N* = 101 (49 in the hungry condition and 52 in the sated condition). Values in *y*-axis are centimeters.

Boxplots in **Figure 1** for VAS-measurements also confirm that the participants felt hungry when arriving at the experiment. None of the results for pre-treatment VAS-measurements significantly differ between treatment and control conditions. In contrast, post-treatment differences in self-reported hunger- and satiety-related sensations are all significantly different (two-tailed *t*-test of means, *p* < 0.001) between treatment and control conditions. However, thirst levels shown in **Figure 1** are statistically indistinguishable across treatment and control conditions. We conclude that the treatment and control conditions were statistically indistinguishable from each other on pre-treatment blood glucose concentration levels and hunger- and satiety-related sensations. After treatment, the sated treatment group reported a significant decrease in hunger and this could be verified from their blood glucose concentration.

Each participant was randomly assigned to either a sender or trustee role in the trust game so we have on average 1.5 observations per participant (see section “Method”). In total, we have 101 individuals playing *X* = [0,3] rounds as sender and 3-X rounds as receiver. Senders have 101 × 3 = 153 decisions and receivers 130 decisions as in 23 decisions by senders did not send any ECUs. When we aggregate decisions over participants, we have 87 individuals with strictly positive number of sender role rounds and 81 individuals with strictly positive number of receiver role rounds.

**FIGURE 2:**
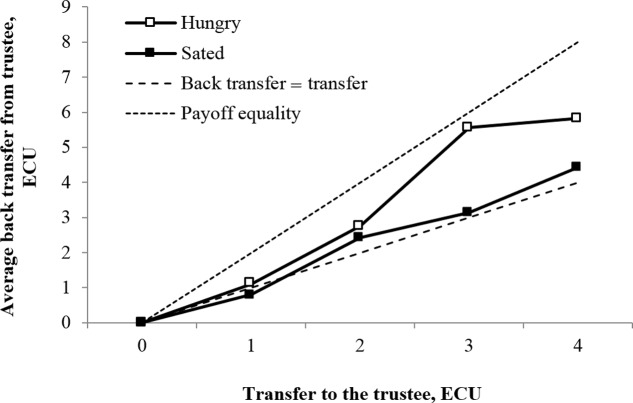
Distribution of back transfers from the trustee in the trust game reported separately for treatment (sated) and control (hungry) conditions. *N* = 130 (*N*_sated_ = 66, *N*_hungry_ = 64).

We report results for trustworthiness in **Figure 2** by replicating the graph from Kosfeld et al. (2005) for back transfers from the trustee to the sender. **Figure 2** indicates that hungry individuals send more funds back at all transfer levels (1–4 ECUs). The difference between the hungry and sated conditions is particularly pronounced in the highest transfers of 3–4 ECUs. At 3 ECU transfers, the trustees in hungry condition almost reach payout equality voluntarily sharing the tripled transfer. At 4 ECUs, this tendency levels off slightly.

On all transfer levels in both the hungry and the sated conditions, trustees on average send back more than what they were initially sent. They did, however, keep a larger share of the additional funds to themselves paid by the experiment organizer consistent with earlier literature (e.g., Kosfeld et al., 2005).

In **Table 2**, we formally report the difference in back transfers between the hungry control and sated treatment conditions. The results for both absolute value of back transfers in Panel A and relative value of back transfers (back transfer/transfer) in Panel B would hint that hungry individuals act more trustworthy than sated individuals, indicating a higher-degree of reciprocity when hungry. On average, a hungry individual sends back 4.17 ECUs compared to 2.89 ECUs by individuals in sated condition. The mean relative back transfer is 142.58% (median 150%) in the hungry condition and 109.97% (median 100%) in the sated condition.

**Table 2:** Back transfers in the trust game.

Panel A: Descriptive statistics on trust experiment, absolute back transfer(*N* = 130 back transfer decisions)		

	Hungry condition	Sated condition
Mean of back transfer (ECU)	4.17	2.89
Median of back transfer (ECU)	4.00	2.50
Standard deviation of back transfers (ECU)	3.17	2.47
Number of observations	64	66

**Panel B: Descriptive statistics on trust experiment, relative back transfer(%) (*N* = 130 back transfer decisions)**		

	**Hungry condition**	**Sated condition**

Mean average back transfer (%)	142.58	109.97
Median average back transfer (%)	150.00	100.00
Standard deviation of back transfers (%)	87.67	78.78
Number of observations	64	66

**Panel C: Mann–Whitney *U* test for difference in absolute mean of transfer(*N* = 8 sessions averaging over 101 individuals with 0–3 back transferdecisions each)**		

	**Test–Statistic**	***p*-value**

Mann–Whitney *U* test *Z*-value	0.29	0.77

**Panel D: Mann–Whitney *U* test for difference in relative mean of transfer(*N* = 8 sessions averaging over 101 individuals with 0–3 back transfer decisions each, 81 individuals with positive number of back transferdecisions)**		

	**Test–Statistic**	***p*-value**

Mann–Whitney *U* test *Z*-value	0.44	0.66


*This table reports results for back transfers from the trust game experiment from a sample of 101 individuals. Panels A and B report descriptive statistics. Panel C reports results for the difference in absolute mean back transfer after first averaging over participants. Panel D reports the corresponding statistics for relative back transfers. All *Z*-values are reported for a two-tailed test.*

We next average across sessions to account for potential clustering of observations at the experimental session level in Panels C and D where we report Mann–Whitney *U* test *Z*-values for the difference between hungry and sated condition. Mann–Whitney *U* test *Z*-statistics on panels C and D are not significant, which is unsurprising given the sample size (*N* = 8). If we assume observations are independent within session but not within individuals Mann–Whitney test yields *Z*-statistics of 2.18 (*p* = 0.03, *N* = 81) for absolute and 1.65 (*p* = 0.10, *N* = 81) for relative back transfer. Taken together, we fail to the reject null hypothesis of no effect when first averaging across experimental sessions. We also cannot rule out the possibility on even having the wrong sign, and as a result of low power, the reported effect sizes should be interpret even more cautiously (Gelman and Carlin, 2014).

We now report in **Table 3**, results for senders in three rounds of trust game played by 101 participants with 153 decisions and 87 participants who played at least one round as a sender. The results in Panel B indicate that individuals in the hungry condition send more money to the trustee than individuals in the sated condition (2.56 ECUs in the hungry control versus 2.19 ECUs in the sated treatment condition). However, the trust game sender decisions in Panel B of **Table 3** have smaller differences between hungry and sated condition than receiver results in **Table 2** (mean transfer for hungry condition 2.58 vs. 2.26 for sated condition after averaging on session level, *Z*-value of 0.29, *p* = 0.77, *N* = 8; *Z*-value of 1.1, *p* = 0.27, *N* = 87 without averaging) and hence do now allow for robust inference. However, setting the issue of lack of statistical significance aside for a moment, the difference between the results for trust game sender and receiver would be in line with Rand (2016). Receiver plays a game of pure cooperation, whereas sender plays a game of strategic cooperation. For the receiver pure cooperation role, short-term hunger matters more, in line with the argumentation in Rand (2016).

**Table 3:** Mean transfer for treatment (sated) and control (hungry) groups.

Panel A: Descriptive statistics on trust experiment transfer (*N* = 153transfer decisions)		

	Hungry condition	Sated condition
Mean of transfer (ECU)	2.56	2.19
Median of transfer (ECU)	2.00	2.00
Standard deviation of transfers (ECU)	1.40	1.38
Number of observations	75	78

**Panel B: Mann–Whitney *U* test for difference in relative mean of transfer(*N* = 8 sessions averaging over 101 individuals with 0–3 transfer decisions each, 87 individuals with positive number of transfer decisions)**		

	**Test–Statistic**	***p*-value**

Mann–Whitney *U* test *Z*-value	0.29	0.77


*This table reports results for transfers from the trust game experiment from a sample of 101 individuals. Panel B reports results for the difference in mean absolute transfer after first averaging over participants. All *Z*-values are reported for a two-tailed test.*

**FIGURE 3:**
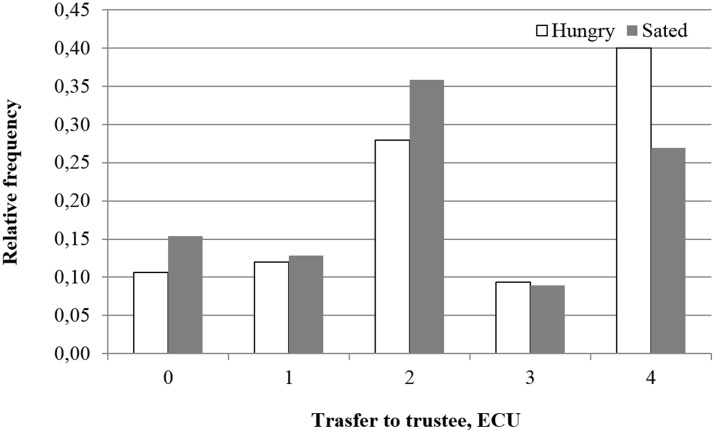
Distribution of transfers to the trustee in the trust game reported separately for treatment (sated) and control (hungry) conditions. *N* = 153 (*N*_sated_ = 78, *N*_hungry_ = 75).

We also investigate in **Figure 3** the more granular distribution of decisions by the sender. The most significant differences in the decision to send funds to the trustee originate from whether to send the highest possible value (4 ECUs).

Although fail to reject the null hypothesis for both trusting and reciprocating when conservatively using experimental session as the unit of observation, the evidence would not be inconsistent with the idea that hungry individuals trust more than sated individuals and the effect magnitude is stronger for trustworthiness.

#### Results for Other Games

Results using normalized measures for all games along with results for the pure cooperation metric are reported in **Table 4**. We report all results averaging across individuals to maintain consistency to results reported published in Rand (2016) and by relaxing the baseline assumption that observations are not independent within experimental sessions.

**Table 4:** Standardized results for all games.

	Hungry condition	Sated condition	*N*	Difference	*t*-stat (2-tailed)	*p*-value
Panel A: Social dilemma						
Mean charitable giving	0.45	0.42	101	0.03	0.40	0.69
Panel B: Pure cooperation						
Mean prisoner’s dilemma (PD)	0.46	0.24	101	0.22	2.95	0.004
Mean PGG	0.80	0.81	101	-0.01	-0.017	0.92
Mean trust game receiver (TGP2)	1.40	1.07	81	0.33	1.84	0.07
Pure cooperation [(PD, PGG, TGP2) as in Rand, 2016	0.65	0.55	101	0.11	2.08	0.04
Panel C: Strategic cooperation						
Mean trust game sender (TGP1)	0.64	0.56	87	0.08	1.11	0.27


*Panel A reports aggregated results for two charitable giving decisions (Red Cross Disaster Relief Fund and New Children’s hospital), Panel B for decisions involving pure cooperation (see e.g., Rand, 2016), and Panel C a decision of strategic cooperation (trust game sender). Pure cooperation metric (reported in Figure 2 of Rand, 2016) combines individual decisions from prisoner’s dilemma, public good game, and trust game receiver role. All statistics are aggregated on individual level from three rounds of games in laboratory. All results are standardized. In Panel A, 1 indicates full contribution of endowment. In Panel B, 1 indicates cooperation in Prisoner’s dilemma (0 for defect), 1 full endowment contribution in public good game (0 for zero contribution), 1 sending back original endowment (before multiplication, 0 for sending back nothing) in trust game. In Panel C, 1 indicates sending full endowment in the trust game.*

The results in **Table 4** would seem to be consistent with the SHH. Hungry subjects are more cooperative in PD (46% vs. 24% cooperation, *t*-value 2.95, *p* = 0.004) and sending back funds in the trust game (140% vs. 107% back transfer, *t*-value 1.84, *p* = 0.07). However, the PGG results are at first glance at odds with the SHH (80% vs. 81% original endowment contribution, *t* = –0.11, *p* = 0.92). The large base rates in the PGG may bear the answer: a highly-automated decision with low cognitive conflict will show up with extreme responses (either close to 0 or 1) and such decisions would be least prone to cognitive load manipulation, such as hunger. This interpretation would be consistent with the models in Bear and Rand (2016) and Bear et al. (2017) in which an agent will switch between intuitive and deliberative responses depending on the deliberation cost threshold, *T*. The dominant strategy (e.g., intuitively defecting or intuitively coordinating) is a function of the probability of the coordination by the other player in the model of Bear et al. (2017). The closer this probability is to 1, the more likely that intuitively coordinating is a good strategy for reasonable other parameter estimates in the model. Alternatively, subjects could have misunderstood the PGG which would explain the failure to reject null in favor of the SHH. This second potential explanation would be consistent with Strømland et al. (2016) who reanalyze evidence on SHH and conclude that participant failure to understand PGG reconciles some conflicting evidence in the literature.

For charitable giving we find similarly no difference between the hungry vs. sated condition (45% vs. 42% charitable giving, *t*-value 0.40, *p* = 0.69). Failing to reject null for altruism is consistent with Hauge et al. (2016) and Tinghög et al. (2016) who do not find evidence on cognitive load having an impact on altruism in experimental settings similar to ours. The results would also line up with Kessler and Meier (2014) who suggest that cognitive load may be differently effective early and late in a session for charitable giving decisions—the charitable giving was last decision of each round in our laboratory session. However, all of these interpretations for the games other than the trust game must be taken with extreme caution as we did not hypothesize on these games *ex ante*.

### Hidden Experiment for Short-Term Hunger and Trust in the Laboratory

#### Method

This analysis was carried out with the same sample of respondents (*N* = 101) as the trust game. Before participants left the first laboratory room with blood glucose tests and surveys behind them, they were given the choice to either leave their belongings in the first laboratory room or take their belongings with them to the second laboratory room. We report results for this hidden experiment in **Table 5**.

**Table 5:** Hidden experiment for trusting behavior.

Panel A: Descriptive statistics		

	Hungry condition	Sated condition
% leaving personal belongings including valuables	20%	4%
Standard deviation of transfers (incl. valuables)	0.41	0.19
% leaving personal belongings excluding valuables	37%	13%
Standard deviation of transfers (excl. valuables)	0.49	0.34
Number of observations	49	52

**Panel B: OLS-regression results for personal belongings includingvaluables**		

Coefficient for sated condition		–0.17
*p*-value, wild cluster bootstrapping at session level		0.35
*p*-value, standard errors not clustered		0.01
*R*^2^		0.065
Number of observations		101

**Panel C: OLS-regression results for personal belongings excludingvaluables**		

Coefficient for sated condition		–0.23
*p*-value, wild cluster bootstrapping at session level		0.14
*p*-value, standard errors not clustered		0.01
*R*^2^		0.073
Number of observations		101


*Individuals were surveyed at the end of the experimental session on whether they left their personal belongings in the first laboratory room while participating in the game session in the second laboratory room. Panel A shows descriptive statistics for leaving belongings in the first laboratory room. Panel B reports results from individual decision to leave any belongings (including valuables) using a univariate OLS-regression with wild cluster bootstrapping of standard errors at session level (1000 replications). Panel C reports results from individual decision to leave personal belongings excluding valuables similar to Panel B. Number of clusters in Panels B and C is 8.*

#### Results and Discussion

Results in Panel A of **Table 5** corroborate our findings for trust in **Table 3**: individuals who were in the hungry condition are more likely to leave their belongings (indicated by self-reported to a short survey at the end of laboratory session, see end of section “Method”) behind than individuals in the sated condition. In the hungry condition, 20% left their valuables and 37% any items (including large non-valuable items such as jackets and bags) in the first laboratory room. In the sated condition the corresponding values are 4% for valuables and 13% for all items.

We estimate a parsimonious OLS regression with the binary decision to leave belongings in the first laboratory room in LHS and with a dummy for sated condition and an intercept in the RHS using wild cluster bootstraps (Cameron et al., 2008) with 8 clusters and 1000 iterations. As reported in Panels B and C, the coefficient for the differences between the hungry control and the sated treatment condition are not statistically significant (coefficient –0.17, *p* = 0.35) for all belongings and for belongings after excluding valuables (coefficient –0,23, *p* = 0.14). When we do not cluster standard errors at session level, difference between hungry and sated group is statistically significant (*p* = 0.01) for all belongings and belongings excluding valuables. However, to be on the conservative side of the interpretation, we cannot reject the null hypothesis in our analysis.

## Conclusion

We develop three alternative hypotheses in this paper as why hunger may have an impact on trust. The overall empirical evidence in this paper is, however, inconclusive: we fail to identify an effect when using conservative clustering of standard errors at session level. When we assume independent decisions within experimental sessions, the results would be broadly consistent with the social heuristics hypothesis (Bear and Rand, 2016; Bear et al., 2017) and the view that both trust and being trustworthy are automated, default-choice responses in our sample operating in a society where fair division of resources and interpersonal trust are strong social norms.

As we had only a limited number of subjects in our study, an obvious next step would be to redo the analysis using a larger sample and number of experimental sessions, perhaps in a context when not trusting and reciprocating is the norm. In addition, we also have limited understanding the impact of other visceral factors in mediating trust. Thirst, which were controlled for^5^ but not the focus of the present study, would be a prime candidate for an analysis of trust similar to this study. We also had one treatment and one control condition in the laboratory. It would be a natural next step to investigate behavioral responses of individuals in more sharply identified categories of satiety. A study using light meal and *ad libitum* meal manipulations, compared with a control group, could shed light on how important the amount of energy intake on trust and reciprocity is.

## Ethics Statement

This study was carried out in accordance with the recommendations of Aalto University Research Ethics Committee on March 6, 2014 (4.2. §2014_2_Nälkä_ päätöksenteossa KTT Elias Rantapuska, Aalto BIZ) with written informed consent from all subjects (Appendix A4). All subjects gave written informed consent in accordance with the Declaration of Helsinki. The protocol was approved by the Aalto University Research Ethics Committee.

## Author Contributions

ER: hypothesis development, research design, laboratory protocol design, data collection, data analysis, writing manuscript. IJ: hypothesis development, research design, manuscript editing. RF: hypothesis development, research design, laboratory protocol design, data collection, manuscript editing. KH: hypothesis development, research design, laboratory protocol design, data collection, data analysis, writing manuscript.

## Conflict of Interest Statement

The authors declare that the research was conducted in the absence of any commercial or financial relationships that could be construed as a potential conflict of interest.

## Footnotes

1 We use the qualifying term “short-term” for hunger to mark a distinction between short-term craving of food (defined as “an uneasy sensation occasioned by the lack of food” by Merriam-Webster online dictionary) and chronic malnutrition (“a weakened condition brought about by prolonged lack of food”). The definition of hunger “*has historically been used to describe a biological state of acute energy deprivation or subjective state presumably reflecting an actual or impending state of energy deprivation*” (e.g., Lowe and Butryn, 2007). In this paper, we consider subjects who are deprived of breakfast to experience short-term hunger. We verify this deprivation empirically by using blood-glucose concentration measurement and subjective feeling of hunger using visual analog scale (VAS).

2 The meal provided 76 g carbohydrates (59% of total energy, E%), 17 g (28E%) fat and 16 g (12E%) protein. The energy and nutrient content of the sandwich was calculated using the Finnish Food Composition Database Fineli^®^ (http://fineli.fi/index.php?lang$=$en). For the yogurt and juice, we used the energy content information given by the manufacturers. Apart from random variation in meal serving sizes, the energy content of the meal did not vary between participants.

3 A reader worried about *post hoc* hypothesizing (see e.g., Kerr, 1998) may be comforted by the fact that in a grant application submitted to KAUTE Foundation on February 14, 2014 and our research proposal submitted to Aalto University Research Ethics Committee on March 6, 2014 we unambiguously state that we will study the relationship between short-term hunger and trust and do not hypothesize on other outcome variables. Furthermore, we also explicitly chose to have participants to complete the trust game first and included hidden experiment to collect further data on trust. Planning to include hidden experiment in our analysis can be confirmed by Appendix 1 of our proposal to Aalto University Research Ethics Committee. Both documents are in Finnish and available upon request from the corresponding author. Our logic proved to have merit *ex post*. Rand (2016) reports results that we did not include in the earlier versions of the manuscript, but were provided to the meta-analysis of Rand (2016) by the authors. In addition, during the review process a referee requested us to add the results for other games in Section “Results for Other Games”.

4 The large fraction of females in the study is most likely attributable to the proximity of the PCRC lab with the faculty of Humanities, where 72% of the student body comprised of females when this study was done.

5 As described in Appendix A, we provide water *ad libitum* to ensure participants are not thirsty. All experimental sessions were scheduled to start at the same time to control for systematic diurnal patterns in fatigue across treatment and control conditions.
