# Supplementary material for: Does Short-Term Hunger Increase Trust and Trustworthiness in a High Trust Society?
Source: Front Psychol. 2017 Nov 7;8:1944. doi: 10.3389/fpsyg.2017.01944 (PMC5681949; doi:10.3389/fpsyg.2017.01944)
Supplement: Supplementary file 1 [file Data_Sheet_1.pdf]

Appendix A1: Detailed description of laboratory procedure

Call for participation

In our call for participation (Appendix A2), we stipulated that the participants must be 18 to 55 years of age, in good health, and be willing to undertake two capillary blood glucose measurements. The exclusion criteria were any metabolic disease (e.g., diabetes), cardiovascular disease, celiac disease, severe lactose-intolerance, relevant food-allergy (citrus, cereal, and milk), eating disorders, strict diet (e.g., vegan diet), or substance abuse. In addition, pregnant or breastfeeding women were excluded from the study. These exclusion criteria were also verified in the laboratory by participants signing informed-consent forms before the start of the experiment. In the call for participation and a reminder sent 24 hours in advance, potential participants were asked to adhere to an overnight fast by not eating or drinking anything other than water 10 hours before the experiment.

The call for participation contained information on a baseline show up fee of 10 EUR in cash and a possibility to win an additional 10 EUR in cash during the experimental session depending on the participant's own and other participants' choices. We also informed subjects that we would serve them breakfast. Finally, the call for participation mentioned that the participants would receive information on their own fasting blood glucose concentration.

First laboratory room (60 minutes)

We have a between-subjects (treatment and control conditions) research design. Each session had 6-20 subjects with all individuals in the same treatment condition with sizes of groups balanced across experimental conditions. We had four randomly assigned groups on four days with treatment ("sated condition") consuming a meal and four groups with control condition ("hungry condition") consuming only water at this point. The research design was single blind: individuals did not know the outcome variable of interest or the condition they were assigned to.

All subjects arrived promptly at the first laboratory room and all experiments began at 09:30.[1] Between 09:30-09:40, participants were briefed on the experiment both verbally and in writing (Appendix A3), and were asked to complete informed consent forms (Appendix A4). The briefing included a general description of how the session would proceed and details of the games in the second laboratory room, including the trust game. The participants received a participant number by drawing a random number between 1-20 unique to each participant. The subjects were asked to write the participant number on each document during the experiment and disclose the participant number to the nurse administering the blood glucose measurement. The participant number also determined seating in the second laboratory room.

At 09:40, the participants rated their hunger and satiety-related sensations (hunger, fullness, satiety, desire to eat, and prospective consumption) and thirst using 10 cm visual analogue scales (VAS, see Blundell *et al*., 2010 and Appendix B1). Then, an experienced nurse measured capillary blood glucose concentrations between 09:40 and 09:50. The sample was taken from the fingertip puncture by sterile single-use lancets (Accu-Check Safe-T Pro plus, Roche Diagnostics, UK). The blood glucose measurement was carried out instantly (Contour® blood glucose monitoring system, Bayer Healthcare, USA). All test strips were from the same batch DP3LJ3D02A. The blood glucose measurements were performed in privacy at a close proximity to the first laboratory room.

At 09:50, the participants were served either a meal or water and they were invited to consume it by 10:00. The participants consumed virtually always the offered meal in silence even though we did not explicitly restrict social interaction. The silent behavior of participants is consistent with cultural norms: Finnish adults consider silence as a natural practice even in the presence of other individuals (see e.g., Carbaugh *et al*., 2006).

[1] Each step was scripted with timing. Apart from the starting time of the experiment, all times are approximate with minor variation between experimental sessions.

The meal consisted of a cheese sandwich, yogurt, and orange juice with an average energy content of 521 kcal (2190 kJ).[2] The experimenter served water to participants in all groups to ensure participants were not thirsty.

Previous scholars (e.g., Wang and Dvorak, 2010) have used sugar and diet drinks as a manipulation of energy level. As our experimental manipulation intends to capture the effect of an experienced temporary hunger rather than short-term peak in glucose concentration, we decided to use a balanced meal to make the setting a more natural breakfast *versus* a skipped breakfast comparison. We wanted to ensure post-meal satiety by serving a mixed meal containing protein and fat in addition to carbohydrates. The digestion products of these nutrients (amino acids, fatty acids, and glucose) induce satiety through multiple mechanisms (Harrold *et al*., 2012).

After consuming a meal or water, the participants were instructed to spend 15 minutes until 10:15 to complete a survey with a questionnaire related to another study, a control questionnaire on emotional valence, and a second VAS-assessment at the end of the survey (Appendix B2).

The nurse measured blood glucose concentrations for the second time between 10:15-10:25, 25 to 35 minutes after starting the meal or drinking water. The participants returned briefly to the first laboratory room to finalize a lottery related to the questionnaire of another study. Next, the experimenter asked participants to proceed to the second laboratory room and casually mentioned "you can leave your belongings such as jackets and bags here or take them with you, it is entirely up to you." This is a hidden setup for an additional investigation of trust for Study 1B. At 10:30, the participants walked into the second laboratory in the same building.

From the initial sample of 108 participants, we have data on 101 individuals. Following our research design approved by the Research Ethics Committee of the university of the corresponding author on March 6, 2014 prior to the laboratory experiments, 6 individuals were instructed by the

[2] The meal provided 76 g carbohydrates (59% of total energy, E%), 17 g (28E%) fat and 16 g (12E%) protein. The energy and nutrient content of the sandwich was calculated using the Finnish Food Composition Database Fineli® (http://fineli.fi/index.php?lang=en). For the yogurt and juice, we used the energy content information given by the manufacturers.

experimenter to discontinue the experiment due to low fasting blood-glucose concentration (<4.0 mmol/L).[3] Furthermore, one individual was instructed to discontinue after the second blood-glucose measurement.[4] These participants were served breakfast immediately and their blood glucose concentration was measured again after 15 minutes to ensure it had increased above 4 mmol/L. We erred on the side of extreme caution on individual health concerns by excluding these 7 individuals from the experiment.

Second laboratory room (20 minutes)

The trust game (see Berg, Dickhaut, and McCabe, 1995) is perhaps the most important research tool in experimental social sciences for measuring trusting and trustworthy behavior. In the trust game, the first mover (trustor or sender) faces a choice of whether or not to send money to the second mover (trustee). If the first mover trusts by sending money to the trustee, any money received by the trustee is tripled by the experimenter. The trustee can send back some part of the tripled funds, but can also choose to defect by returning less than what the first mover sent. Both parties are strictly better off when trusting and reciprocating.

We had the trust game as a part of the laboratory session in the second laboratory room (Appendix C). The individuals completed the trust game first, followed by a prisoner's dilemma, a public goods game, and two dictator games with donations to external recipients (for summary descriptions of games, see Levitt and List, 2007).

[3] One individual had fasting blood-glucose concentration of 3.9 mmol/L at first measurement. This individual was prompted for willingness to continue the experiment by the nurse and this participant decided to continue.

[4] As one individual discontinued the experiment after the second blood glucose measurement just before moving into the second laboratory room, we had one nurse sitting in one laboratory session in the second laboratory room to retain an even number of participants in the session. We did this not to cause any disruption and delay in the experimental session resulting from re-starting the experiment software and having to exclude one further participant from the experiment while the experiment was in progress. The participation of the nurse was not made salient to other participants, she was advised to act as she would normally do, she had the same incentives as other participants, and her observations were excluded from all analyses.

Similar to Schotter, Weigert, and Wilson (1994) we were concerned about subjects not perceiving the trust game as a strictly one-off problem and either learning or establishing a norm of future play through their own actions. To alleviate such concerns, we randomized the roles in each round to induce participants to consider each round strictly as a one-off decision and limited the number of standard trust game rounds (described in detail in Berg *et al*., 1995 and Kosfeld *et al*., 2005) to three with a randomly chosen counterpart and role (sender or trustee) redrawn with replacement each round. Although mentioned in the instructions in the first laboratory room, the experimenter reminded participants on randomization before the beginning of the trust game. Each participant would thus play a randomly assigned number (0-3) of rounds of the trust game as a sender and the remaining rounds as a receiver.

We used experimental currency units (ECUs) in the experiment with ten ECUs corresponding to 1 EUR. In each trust game, the sender was endowed with 4 ECUs and could send any number between 0-4 ECUs to the trustee with an initial 4 ECU endowment. Any value of ECUs sent would be tripled and the trustee could now send back any value of ECUs of his or her current endowment which would be 4 ECUs + 3 x (value sent by the sender). We learned from the review of Johnson and Mislin (2011) that deviations from the original 10 USD endowment values in Berg *et al*. (1995) do not systematically impact results across trust games and decided to change the initial endowment to obtain a larger number of observations.

In prisoner's dilemma game, players have to decide whether to cooperate or defect. Players will achieve highest combined payoff (3 ECUs to both) by cooperating, but a player has always an incentive to cheat for a higher payoff. In case the other player cooperates, the defector gets 5 ECUs and the cooperator gets nothing. In case both players defect, the payoffs are 1 ECU to both players.

In public good game, both players receive 4 ECUs which they can partially or wholly contribute to common pool, which is doubled by the experimenter and equally shared between participants. We use pairwise iteration so players will learn the outcome of the other player at the end of each round.

Similar to prisoner's dilemma, both players have an incentive to defect by not contributing to common pool.

In the dictator games, participants were allocated 5 ECUs that they could donate wholly or partially to the New Children's Hospital 2017 project and a further 5 ECUs to be potentially donated to the Finnish Red Cross Disaster Relief Fund. We included additional games to avoid replicating the rather expensive and somewhat invasive (being subject to short-term hunger and two blood glucose tests) laboratory session in case requested by the scientific community.

After completing the experimental session in the second laboratory room, participants filled in a short survey including demographic questions (year of birth and gender), height, weight, and a question on leaving personal belongings in the first laboratory room.

Participants would see other individuals during the experiment, but without knowing exactly which participant they are paired with in the second laboratory room. To prevent any coordination during the experimental session, low walls divide the space between participants in the second laboratory room. The experiment was programmed and conducted with the experiment software z-Tree (Fischbacher, 2007).

After laboratory experiment (10 minutes)

Finally, after collection of all experimental data, the experimenter identified him- or herself and distributed earnings for each participant individually in cash and a lunch voucher after receiving participant signature. The nurse also informed participants individually about their fasting blood glucose concentrations after earnings distribution. If the baseline glucose exceeded 6 mmol/L the participant was advised to consult his or her physician. The hungry control condition participants were now served the same meal as the treatment condition in the first laboratory room. Each experimental session lasted up to one hour and 30 minutes.

References not in the main body of the manuscript

Carbaugh, Donal, Michael Berry, & Marjatta Nurmikari-Berry. (2006). Coding Personhood Through Cultural Terms and Practices Silence and Quietude as a Finnish "Natural Way of Being". *Journal of Language and Social Psychology* 25 (3), 203-220.

Johnson, Noel D., and Alexandra A. Mislin. (2011) Trust games: A meta-analysis. *Journal of Economic Psychology* 32 (5), 865-889.

Harrold, Joanne A., Dovey Terry M., Blundell John E., & Halford Janson C.G. (2012). CNS regulation of appetite. *Neuropharmacology* 63 (1), 3-17.

Schotter, Andrew, Keith Weigert, & Charles Wilson. (1994). A Laboratory investigation of multiperson rationality and presentation effects. *Games and Economic Behavior* 6, 445-468.

Appendix A2: Call for participation

Sign up for a study

We are looking for volunteers to participate in a decision making study organized by [University Name 1] and [University Name 2]. The study takes place in the PCRC laboratory (Arcanuminkuja 3, Building 24) where the participants are requested to fill in surveys and participate in a computerized decision making experiment. During the course of the study, a nurse will take two blood samples from your fingertip. The experimental sessions are organized on weekdays starting at 9:30am and they last a maximum of two hours. Participants are requested not to eat or drink anything other than water for 10 hours before the study (so-called overnight fast). On the eve of the study, participants must restrain from substantial use of alcohol. They should also avoid hard physical strain during the previous evening and in the morning of the study. We recommend that the participants drink a glass of water in the morning prior to leaving for the study. A breakfast is served during the study.

Each participant receives a baseline show-up fee of 10 EUR. In addition to the baseline show-up fee, a participant can win 0-10 EUR depending on their own and other participants' choices as well as chance. In addition to these cash payments, participants also receive a Unica lunch voucher and they are informed of their blood glucose reading after fasting overnight (so-called fasting blood glucose concentration). Travel expenses via public transport are also reimbursed against a receipt.

We are looking for healthy, 18-55 year-old volunteers to participate in the study. You are eligible to sign up as a participant if:

- You do not have any illness that can cause harmful side effects during an overnight fast. Such illnesses are diabetes and other metabolic disorders, diseases which can influence heart, liver, pancreas, stomach or kidney function as well as eating disorders
- You do not have any illness or diet that would restrain you from enjoying a meal served during the experimental session. Such illnesses and conditions are celiac disease, grain allergy, severe lactose-intolerance (low-lactose meal), milk allergy, citrus allergy, vegan or low carbohydrate diet
- You are not pregnant or breastfeeding
- You are not aware of any other factor that could cause you harmful side effects when you participate in a 10-12 hour fast without food and drink (besides water)
- You believe you can handle a fingertip blood test and a 10-12 hour fast

If you are interested in taking part in the study, please reserve a suitable time slot at the PCRC lab. If you have any further questions about the study, please contact:

[Name of the principal investigator]
[Title of the principal investigator]
[Affiliation of the principal investigator]
[Email of the principal investigator]
[Phone number of the principal investigator]

Appendix A3: Information on study for participant

Research sheet 1
Information sheet

1. DECISION MAKING STUDY

This study by [University Name 1] investigates decision making. The study is funded by the university and it has no commercial goals. All your answers are treated in strict confidence. Your answers and decisions in the study will be treated as anonymous observations, i.e., your name will not be attached to your answers at any point of the study. At the beginning of the study, you will receive a participant number (1-20) that will be used for combining the data across separate parts of the study. You can discontinue your participation in the study at any stage without a need to justify your choice. Please read the following instructions very carefully. If you have any questions about the study, please feel free to ask the organizers.

This study comprises two separate experiments: first a survey, and second a computerized task. Before the experiments begin, a nurse will take a blood sample from your fingertip and you are asked to evaluate how hungry you are.

2. FIRST PART OF THE STUDY

In the first part of the study, you will be requested to fill in a survey where you are asked to make decisions on choice problems under uncertainty, answering reasoning questions and evaluating both accuracy of given statements and your own states of feeling. It is very important that you read the instructions on the survey carefully and that you fill in the survey accurately. In the A and B parts of the survey, you will be making choices on lotteries that include real monetary payoffs. Lottery winnings will be paid in cash at the end of the entire experimental session for one randomly selected participant according to the details given in the survey. We will randomly draw one participant number and the participant with the participant number will participate in two randomly selected lotteries, one from part A and one from part B of the questionnaire. Parts C-F of the survey do not include any monetary prizes.

A nurse will take a second blood sample at the end of the first part of the study. The blood samples are used to measure your blood-glucose level. The glucose measurements are not performed in a diagnostic manner. It is, however, possible that healthy participants have blood-glucose levels that are outside the reference value range. If your fasting glucose value is outside the reference value range, the nurse may refer you to a physician for further tests. In such an event, you may also reconsider your participation in the study. If you decide to discontinue the study, the result of your blood-glucose measurement will remain only for your personal use.

3. SECOND PART OF THE STUDY

The second part of the study is conducted using computers in the second laboratory room. You will be presented with four different games, during each of which you will play three rounds. At the beginning of each round, you will be randomly paired with a new counterpart from the group of participants, and you will not be informed about the identity of your counterpart during the three rounds. Your counterparts will thus change randomly throughout the experiment. The experiment includes the use of so-called experimental currency, ECU. 10 ECUs correspond to 1 EUR. We will sum up all the ECUs you have won throughout the tasks, and we will convert the ECUs into real Euros and pay them in cash. For instance, if you have 50 ECUs at the end of the study, you will receive 5 EUR in cash.

In addition to the monetary fees from the tasks, each participant will receive a 10 EUR participation fee. You cannot lose this 10 EUR participation fee during the experiments.

During the second part of the study, you might need to wait for the decisions of the other players. Please wait calmly for other players' choices. In order to keep the flow of the study fluent, please do not start doing other things while waiting, such as checking your mobile phone.

You will be asked for some essential background information at the end of the second part of the study.

4. SECOND PART OF THE STUDY: A DESCRIPTION OF THE GAME SETUPS

The following is a short description of each game setup that you will encounter during the second part of the study.

GAME 1

You can be either the first decision maker ("A") or the second decision maker ("B") in Game 1. Your role is assigned randomly.

If you are the first decision maker ("A") in Game 1:

You receive 4 ECUs from the experimenter. You can send 0-4 ECUs to another player in the room who has been randomly selected to be second decision maker "B". The experimenter triples all the money you have sent to the other player "B". The randomly selected other player "B" decides at the end of the round how much money s/he wants to send back. S/he can also decide to keep all the money you sent. You and the other player cannot communicate with each other.

If you are the second decision maker ("B") in Game 1:

You are player "B". The other player, who is another, randomly selected participant in the room, has 4 ECUs at the beginning of the game. The other player can send you a self-selected amount of ECUs (0-4). The experimenter triples all the money sent to you by the other player "A". Make a decision on how many ECUs you wish to send back to the other player "A". You and the other player cannot communicate with each other.

GAME 2

You are playing the game with a randomly selected participant from the same room. If you both select 1, you will receive 3 ECUs. If you select 1 and the other player selects 2, you will receive 0 ECUs. If you both select 2, you will receive 1 ECU. If you select 1 and the other player 2, you will receive 5 ECUs. You and the other player cannot communicate with each other.

GAME 3

You receive 4 ECUs. You can decide to put 0-4 ECUs into a common treasury where both your and the other player's contributions will be placed. The experimenter will add to the treasury 1 additional ECU for each 2 ECUs that you and the other player have contributed. At the end of the game, the ECUs in the treasury are split equally between you and the other player.

GAME 4A

You receive an extra 5 ECUs. If you want, you can donate 0-5 ECUs to the New Children's Hospital. All donated ECUs will be exchanged for real money and donated to the New Children's Hospital after the experiment.

GAME 4B

You receive an extra 5 ECUs. If you want, you can donate 0-5 ECUs to the Finnish Red Cross Disaster Relief Fund. All donated ECUs will be exchanged for real money and donated to the Finnish Red Cross Disaster Relief Fund after the experiment.

5. THANK YOU FOR YOUR PARTICIPATION!

Thank you for your participation in this study! The study will help us increase the understanding of human decision making. On request, the principal investigator will give you further information on the general principles, progress of the study and the results concerning you personally.

On behalf of the research team,

[Name of the principal investigator]
[Title of the principal investigator]
[Affiliation of the principal investigator]
[Email of the principal investigator]
[Phone number of the principal investigator]

Appendix A4: Informed consent

Research sheet 2: Participant number (1-20):
Informed consent for a decision making study

I have read and understand the content of the Information sheet and I have been sufficiently informed about the course of the study. I am aware that a nurse will take two blood samples from my fingertip for a glucose measurement during the study. I am over 18 years old and I am not aware of any health issues (diabetes or another metabolism disorder, diseases influencing heart, liver, pancreas, stomach or kidney function or eating disorders) that can cause harmful side effects for me during a 10-12 hour fast. I am not pregnant or breastfeeding. I also do not have any illness or other restrictions that influence my diet: celiac disease, grain allergy, severe lactose-intolerance, milk allergy, citrus allergy, other restricting diet (such as vegan or low carbohydrates diet).

I am aware that I can discontinue my participation in the study at any stage without any adverse effects on me. I can also cancel my consent to participate, in which case all the information and samples collected will not be used for research purposes. I have been informed that I can receive further information from the principal investigator on the general principles, progress of the study and the results concerning me personally.

The research findings that concern me personally are only available to the researchers involved in the research project. The principal investigator can, however, give the collected data as a part of a larger data set for analysis and other scientific purposes to other collaborators without any further consent from me. In such an event, the principal investigator ascertains the anonymity of the results and the data.

I understand that the data are collected only for scientific purposes and that other data, besides for the fasting glucose value, will not be given to me personally. By signing this, I confirm my participation in this study as a volunteer participant.

Signature of the participant Date

Printed name Social security number

Address of the participant
