# Supplementary material for: Does Short-Term Hunger Increase Trust and Trustworthiness in a High Trust Society?
Source: Front Psychol. 2017 Nov 7;8:1944. doi: 10.3389/fpsyg.2017.01944 (PMC5681949; doi:10.3389/fpsyg.2017.01944)
Supplement: Supplementary file 2 [file Data_Sheet_2.pdf]

Participant number (1-20):

Appendix B1: VAS

<Date>

Please answer the following questions by marking a cross on the line at the point that best describes your current state (see example below). Please do not let one statement influence your answer on another statement but answer each statement independently.

Not at all ——————X—————— Extremely

1. How hungry are you?

Not at all hungry ________________________________ Extremely hungry

2. How full are you?

Not at all full ________________________________ Extremely full

3. How satiated are you?

Not at all satiated ________________________________ Extremely satiated

4. How strong is your desire to eat?

Very weak ________________________________ Very strong

5. How much do you think you could eat right now?

Nothing at all ________________________________ A very large amount

6. How thirsty are you?

Not at all thirsty ________________________________ Extremely thirsty

This survey is related to a scientific study at [University Name 1]. Your answers will be analyzed anonymously and strictly confidentially.

Participant number (1-20):

Appendix B2: Questionnaire and VAS

<Parts A – D were related to another study currently in progress>

<Beginning of the document includes the text below>

Please answer to all questions in parts A-F.

In parts A and B you can win ECUs depending on your answers. ***ECUs will be translated to real Euros (10 ECUs corresponding to one Euro) and paid in cash for one randomly selected participant at the end of the experiment.*** *In part B, you will start with an endowment of 4 ECUs. In parts C-F there is no money at stake. You can discontinue the experiment any time if you wish. Your answers will be analyzed anonymously. When complete, please hand in the sheet to the experimenter.*

DO NOT USE MOBILE PHONE, (TABLET) COMPUTER, DISCUSSION WITH OTHER PARTICIPANT OR ANY OTHER METHOD TO ASSIST YOUR CHOICE. PLEASE ANSWER ACCORDING TO YOUR BEST EVALUATION AND CHOICE INDEPENDENTLY.

This survey is related to a scientific study at [University Name 1]. Your answers will be analyzed anonymously and strictly confidentially.

PART E

Please answer the following questions by marking a cross on the line at the point that best describes your current state (see example below).

Not at all ——X—— Extremely

E1. How positive do your feel?

Not at all positive ________ Extremely positive

E2. How negative do your feel?

Not at all negative ________ Extremely negative

E3. How calm or aroused do your feel?

Very calm ________ Very aroused

E4. How angry do your feel?

Not at all angry ________ Extremely angry

E5. How sad do your feel?

Not at all sad ________ Extremely sad

E6. How happy do your feel?

Not at all happy ________ Extremely happy

E7. How disgusted do your feel?

Not at all disgusted ________ Extremely disgusted

E8. How frightened do your feel?

Not at all frightened ________ Extremely frightened

E9. How surprised do your feel?

Not at all ________ Extremely

This survey is related to a scientific study at [University Name 1]. Your answers will be analyzed anonymously and strictly confidentially.

surprised ____________________ surprised

PART F

Please answer the following questions by marking a cross on the line at the point that best describes your current state (see example below). Please do not let one statement influence your answer on another statement but answer each statement independently.

Not at all ——————X—————— Extremely

F1. How hungry are you?

Not at all hungry ____________________ Extremely hungry

F2. How full are you?

Not at all full ____________________ Extremely full

F3. How satiated are you?

Not at all satiated ____________________ Extremely satiated

F4. How strong is your desire to eat?

Very weak ____________________ Very strong

F5. How much do you think you could eat right now?

Nothing at all ____________________ A very large amount

F6. How thirsty are you?

Not at all thirsty ____________________ Extremely thirsty
