# Supplementary material for: Does Short-Term Hunger Increase Trust and Trustworthiness in a High Trust Society?
Source: Front Psychol. 2017 Nov 7;8:1944. doi: 10.3389/fpsyg.2017.01944 (PMC5681949; doi:10.3389/fpsyg.2017.01944)
Supplement: Supplementary file 3 [file Data_Sheet_3.PDF]

Appendix C:
Session in the second laboratory room with screenshots

Flow of games in the second laboratory room

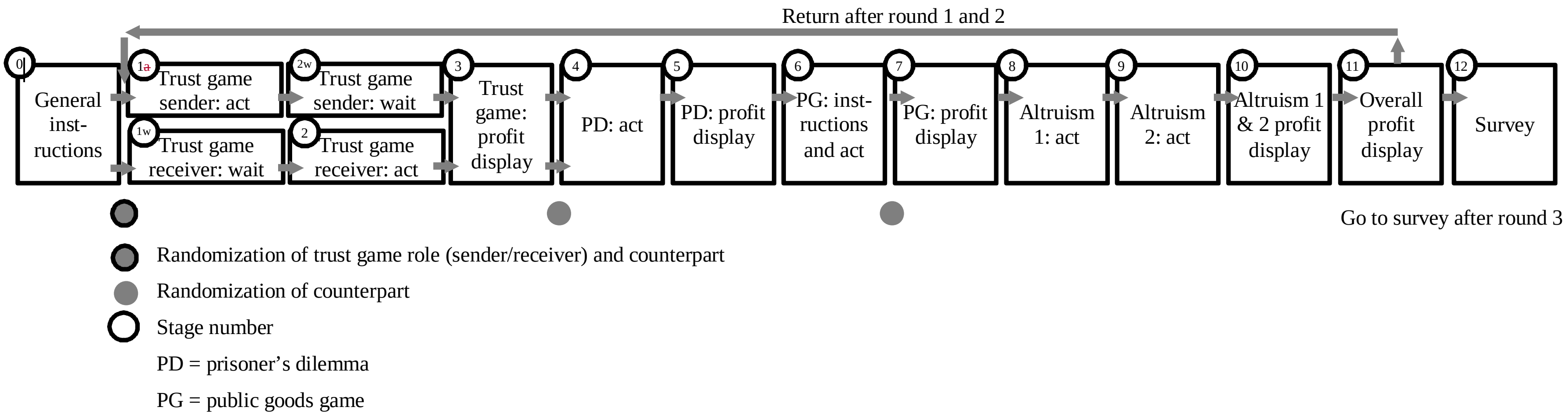

Key to screenshots

The laboratory session was conducted originally in Finnish. This document provides original screenshots with a translation.

○ The number in the top left corner of the page corresponds to the stage of the game. The letter “w” refers to waiting screen shown while the other player(s) is/are making his/her/their decision(s). These screens are only shown if a participant is waiting for a decision by another player(s).

[X] denotes an input parameter supplied by the participant

[Y] denotes a parameter displayed to the participant

The top left-hand corner shows text “round [Y]/3” indicating the order of game session rounds.

The top right-hand corner has a countdown indicator from 90 to 0 in seconds with text “time left to read instructions/time left (in seconds)” with text “please click continue” or “please reach a decision” shown after 90 seconds.

Buttons in the bottom right-hand corner of screenshots with text “Jatka” correspond to “Continue”.

0

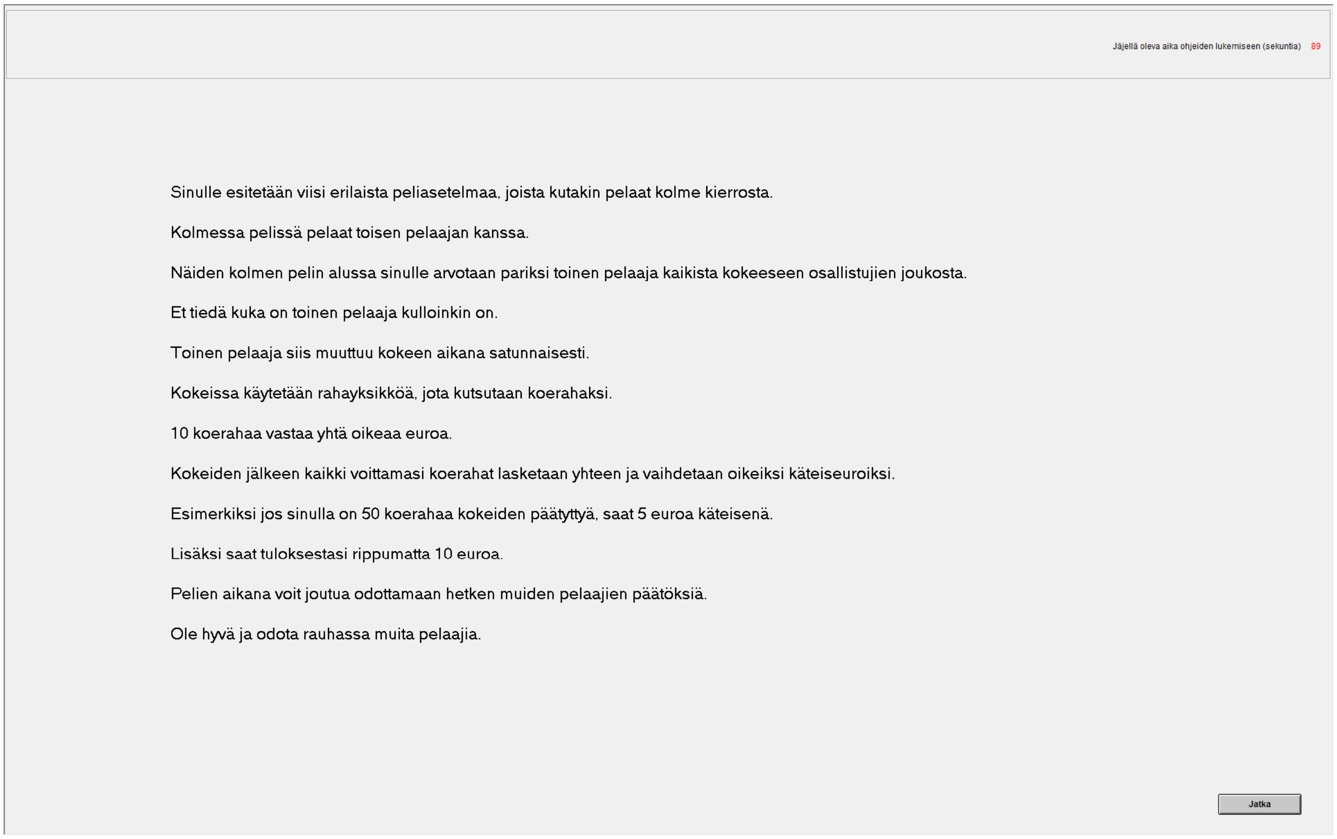

You will be shown five different games, of which you will play each for three rounds.

In three games, you will play with other participants.

At the beginning of these three games, you will be randomly paired with a participant from the group.

You do not know who you are paired with at any given time.

The other player thus changes randomly during the experimental session.

The games use the experimental currency unit, ECU.

10 ECUs correspond to 1 real Euro.

After the experiments, all ECUs will be summed up and exchanged for real cash Euros.

In addition, you will be paid 10 Euros independent of your result.

During the games, you may need to wait a while for decisions by other players.

Please wait patiently for other players.

0w

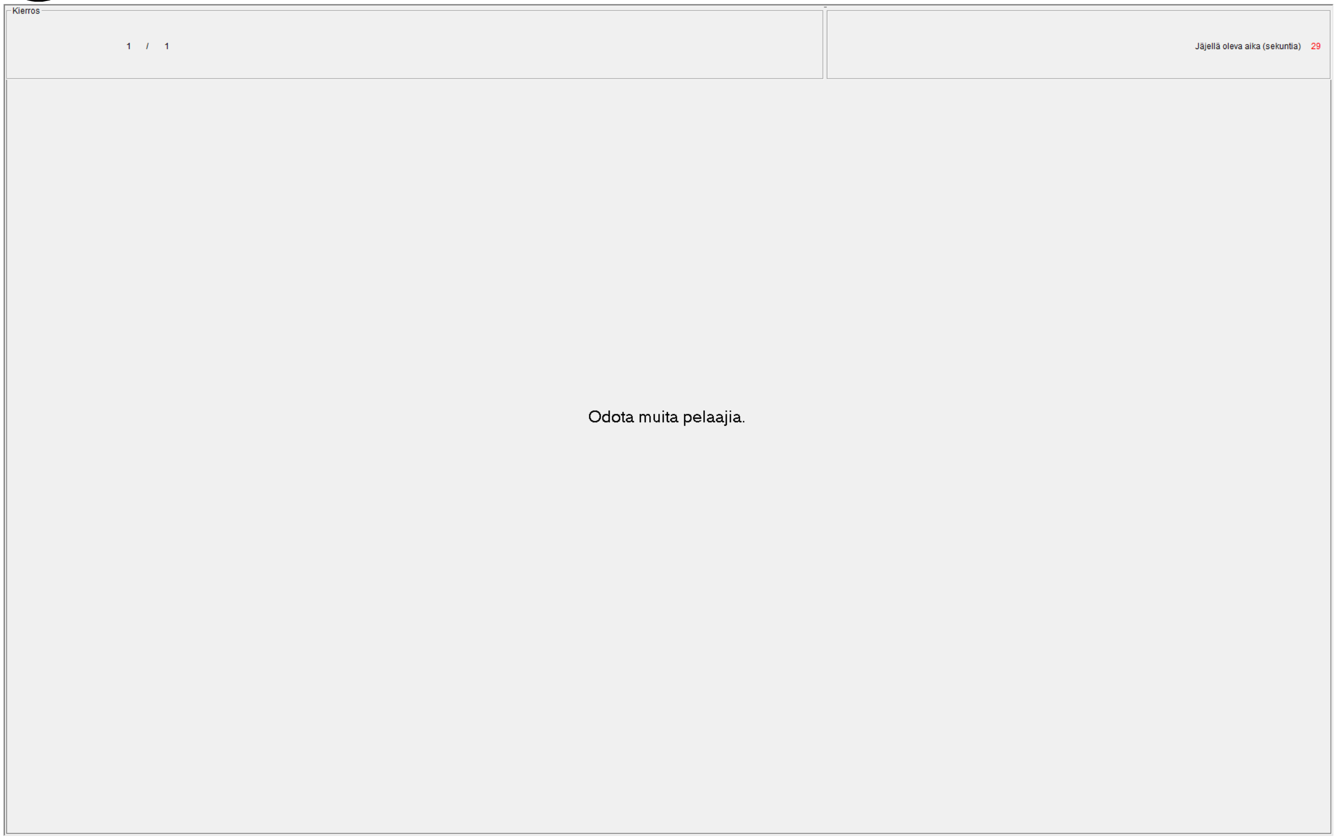

Wait for other players.

1

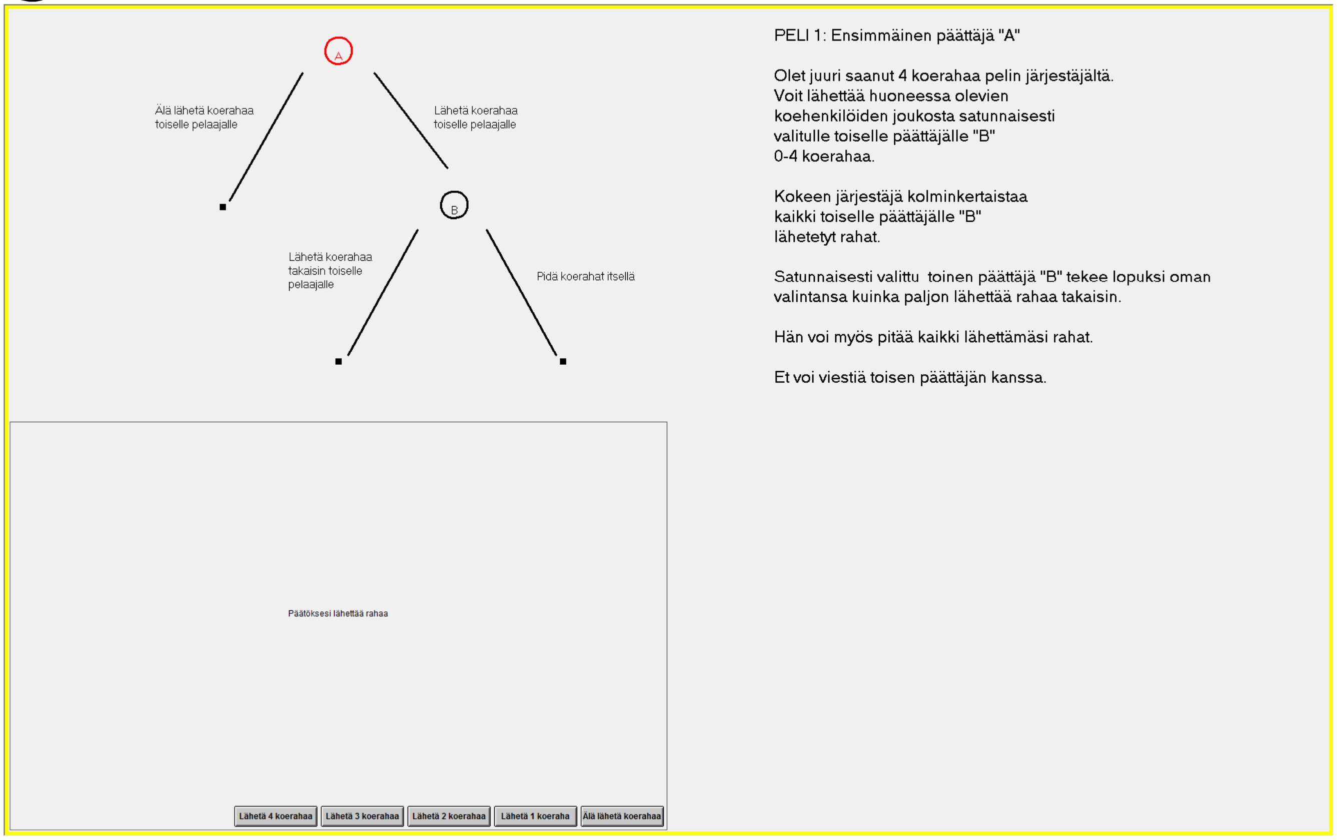

[Text on the left-hand side under (A)]

Do not send ECUs to the other player.

Send ECUs to the other player.

[Text on the left-hand side under (B)]

Send ECUs back to the other player.

Keep ECUs to yourself.

[Text in the bottom-left box]

Your decision to send funds

Send 4 ECUs/Send 3 ECUs/Send 2 ECUs/Send 1 ECU/Do not send ECUs

[Text at top right]

GAME 1: First decision maker “A”

You have just received 4 ECUs from the experimenter. You can send 0-4 ECUs to second decision maker “B” who has been randomly drawn from the group of participants in the room.

The experimenter will triple all funds sent to second decision maker “B”.

Randomly drawn second decision maker “B” will, at the end of the game, make his/her own decision on how many ECUs to send back.

He/she can also keep all the funds you have sent.

You cannot communicate with the second decision maker.

1w 2w

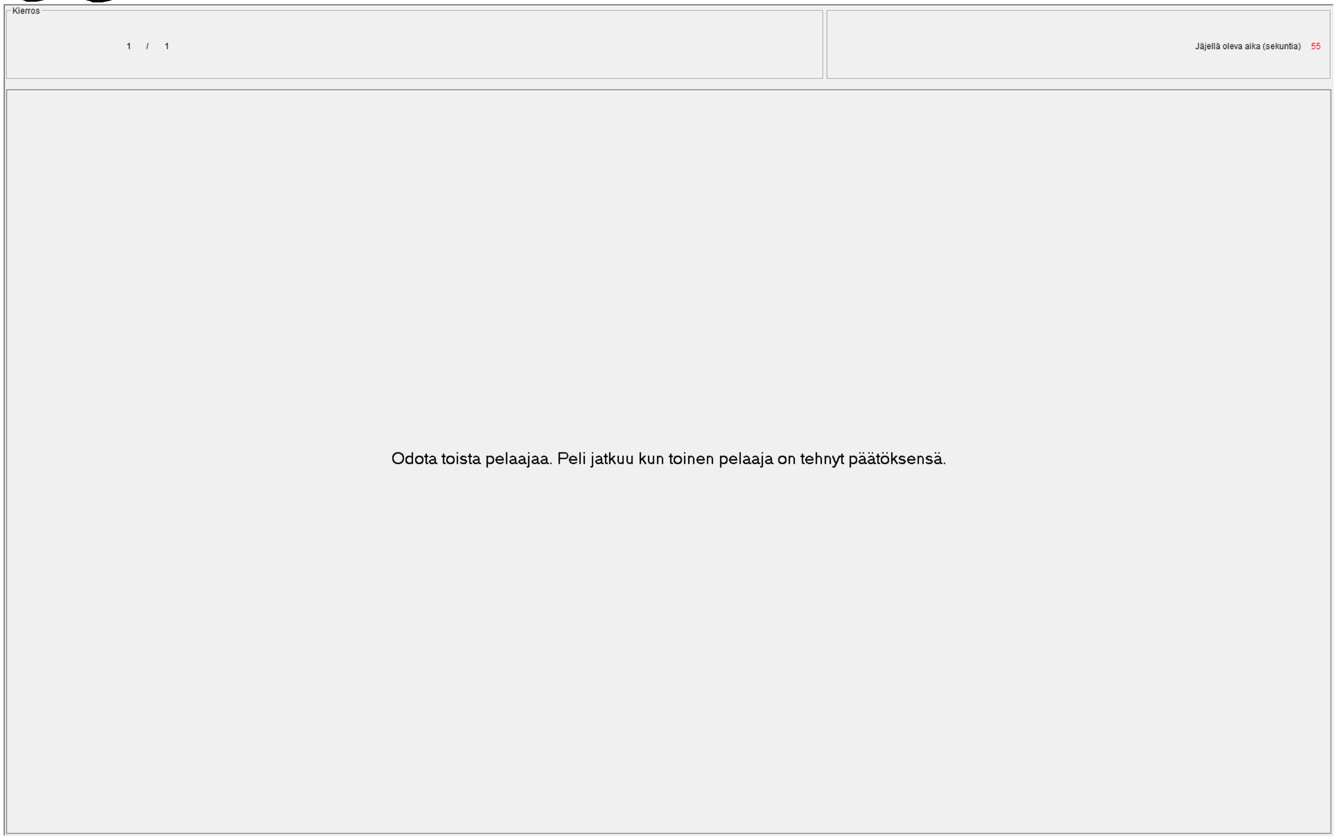

Wait for the other player. The game will resume after the other player has reached his/her decision.

2

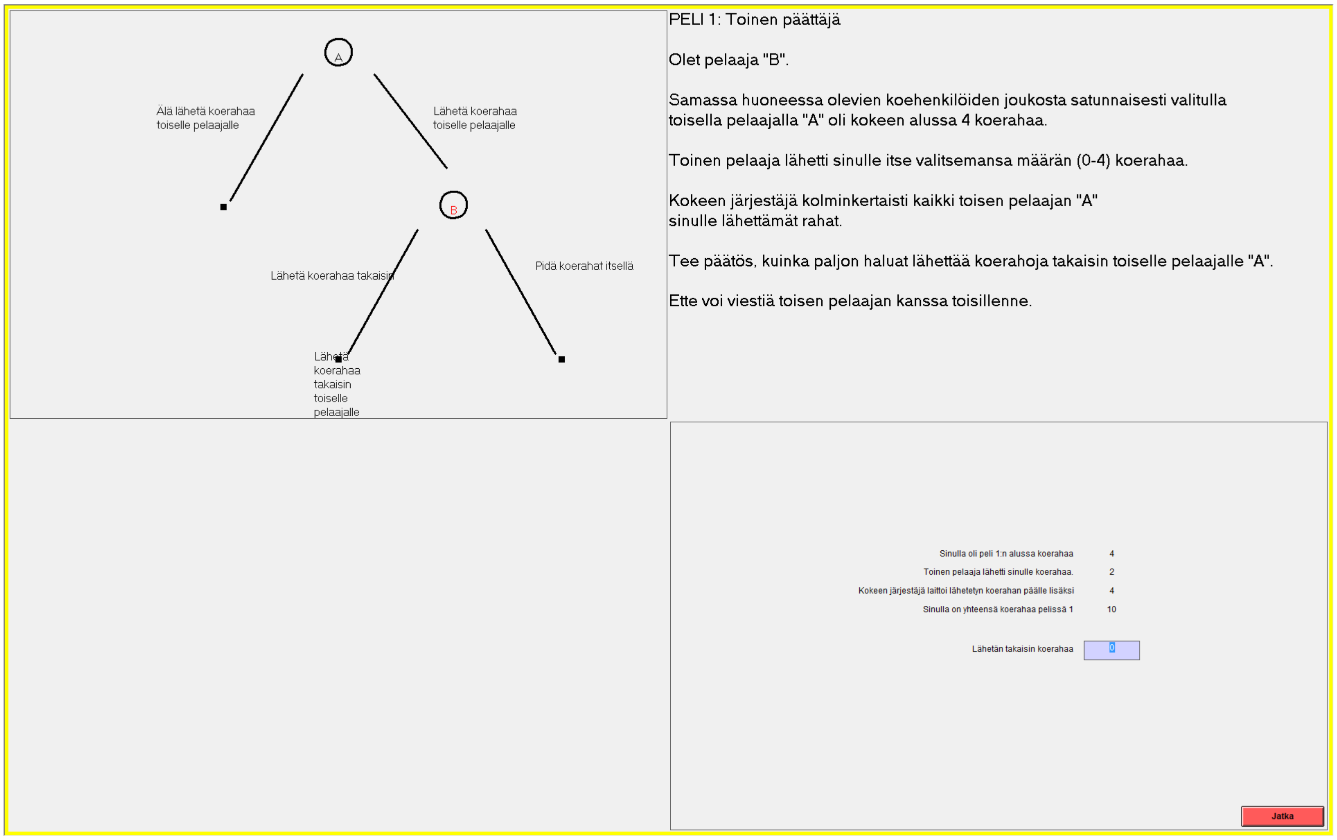

[Text at top right]

GAME 1: Second decision maker

You are player “B”.

The other player, “A”, drawn randomly from the group of participants in the same room, had 4 ECUs at the beginning of the experiment. The other player has sent you an amount of ECUs (0-4) which he/she has decided on him/herself.

The experimenter tripled all funds sent to you by player “A”.

Make a decision on how many ECUs you want to send back to the other player.

You cannot communicate with each other.

[Text on bottom right-hand side, X corresponds to parameters that depend on decisions made]

You had ECUs at the beginning of Game 1:	[Y]
The other player sent you ECUs	[Y]
The experimenter added to sent funds	[Y]
You have a grand total of ECUs	[Y]
I will send back ECUs	[X]

[Text on the left-hand side under (A)]

Do not send ECUs to the other player.

Send ECUs to the other player.

[Text on the left-hand side under (B)]

Send ECUs back to the other player.

Keep ECUs to yourself.

3

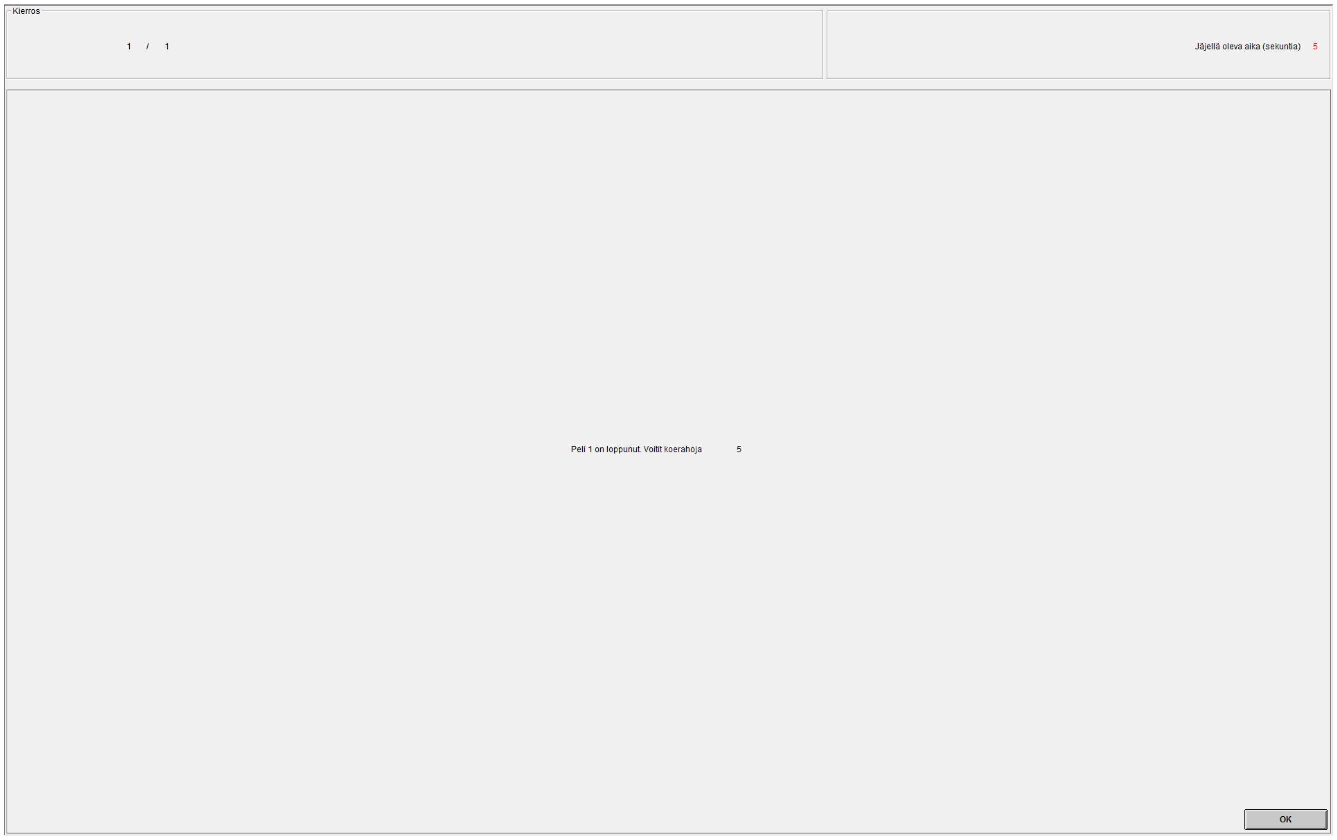

Game 1 has ended. Your profit in ECUs [Y]

4

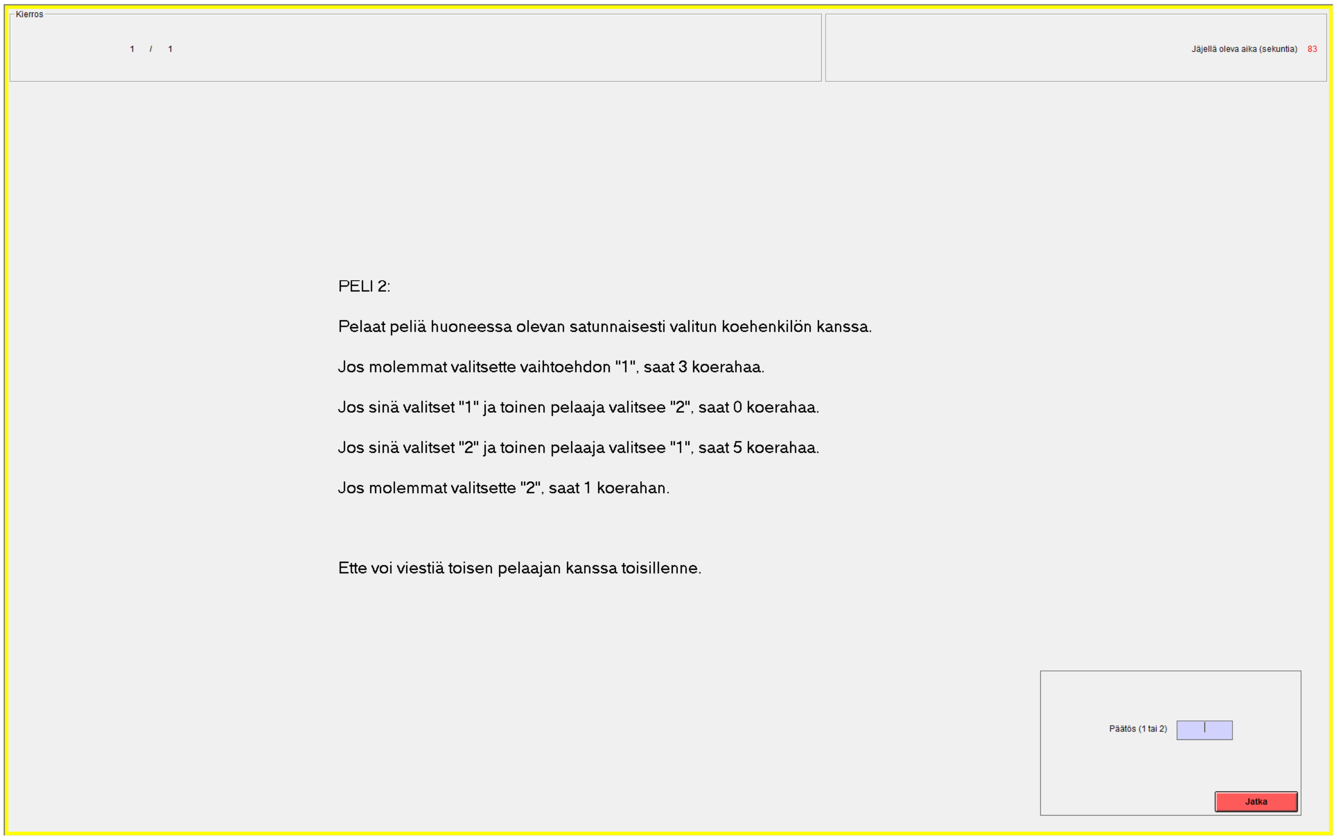

GAME 2:

[Text at top]

You play the game with a randomly drawn participant in the same room. If you both select “1”, you will get 3 ECUs.

If you select “1” and the other player “2”, you will get 0 ECUs.

If you select “2” and the other player “1”, you will get 5 ECUs.

If you both select “2”, you will get 1 ECU.

You cannot communicate with each other.

[Text on bottom right-hand side]

Decision (1 or 2) [X]

4w

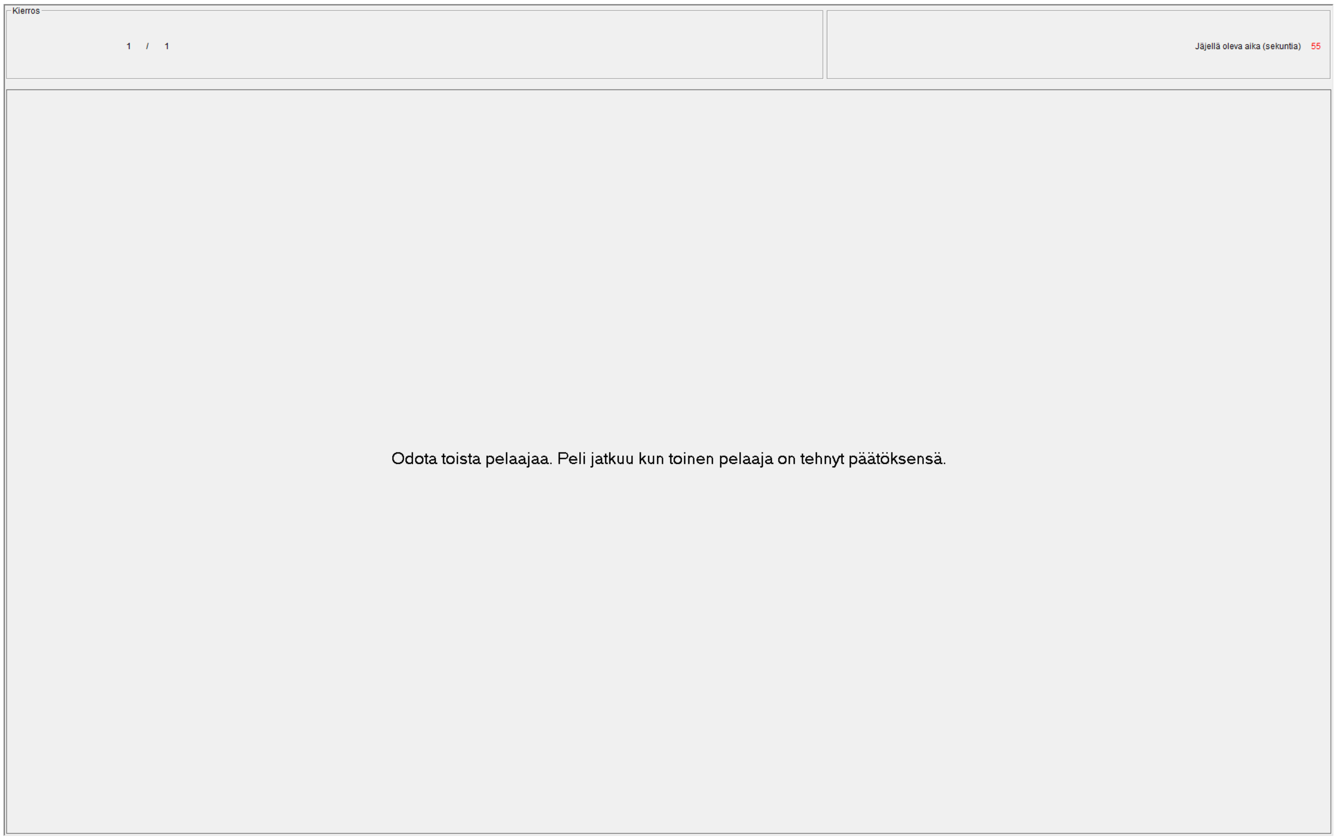

Wait for the other player. The game will resume after the other player has reached his/her decision.

5

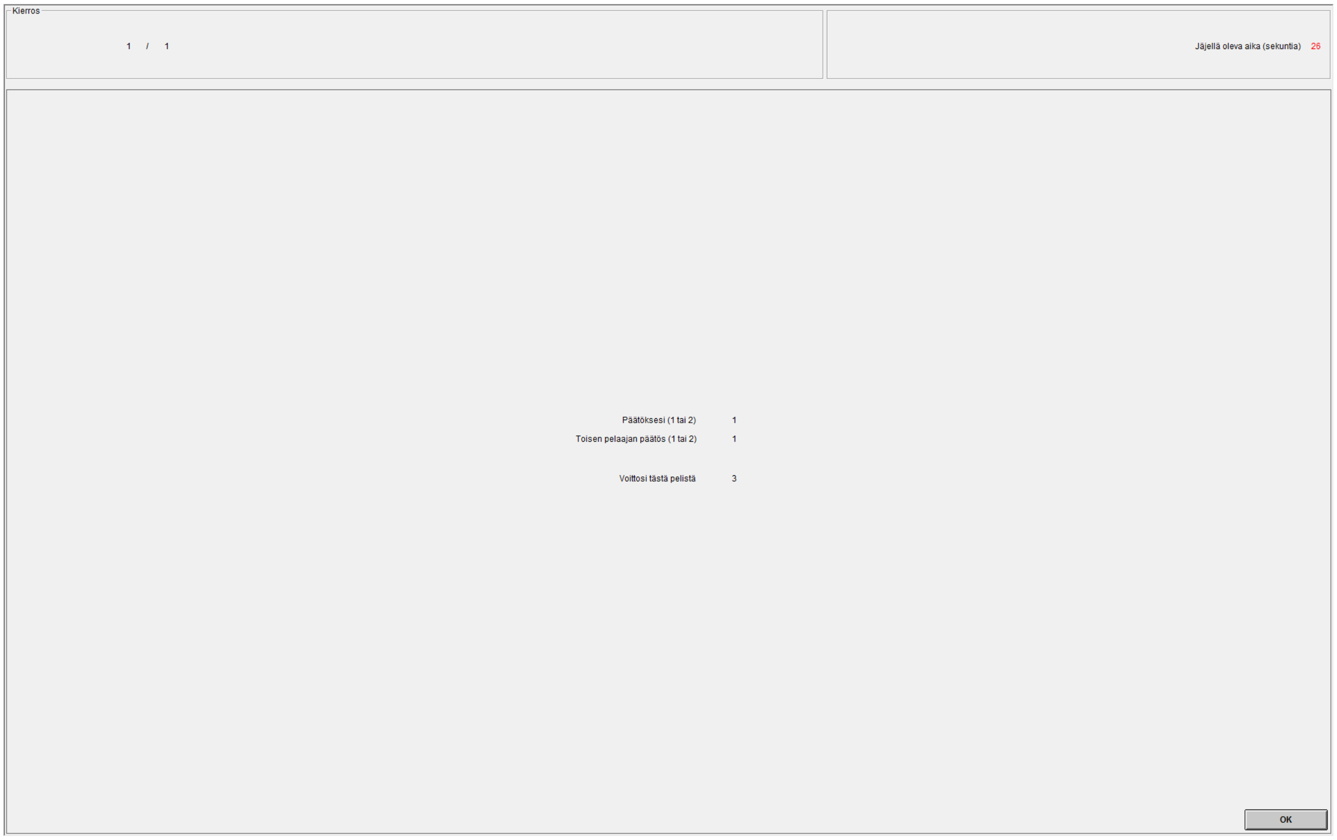

Your decision (1 or 2)	[Y]
Decision by the other player (1 or 2)	[Y]
Your profit from this game	[Y]

6

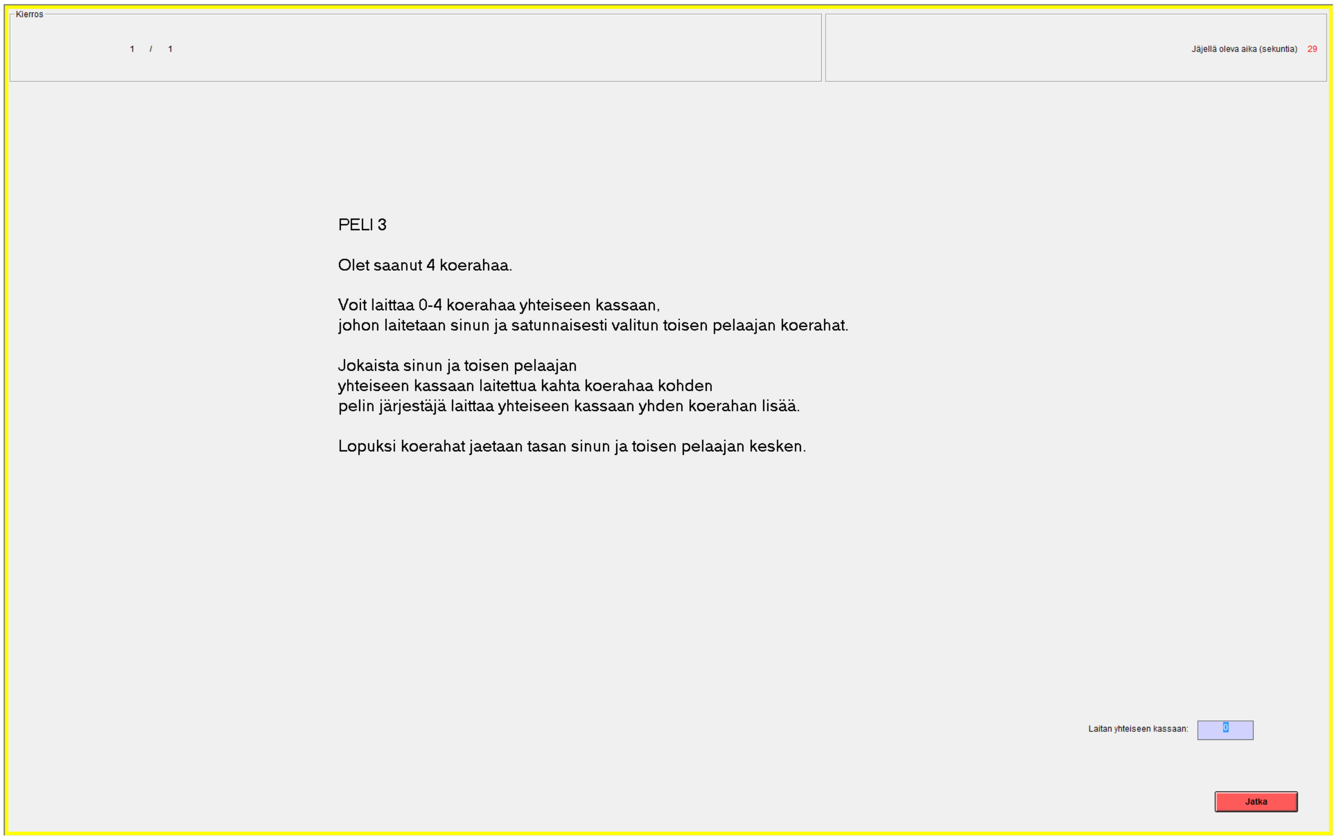

[Text at top]

GAME 3

You have received 4 ECUs.

You can contribute 0-4 ECUs to common treasury, which will contain contributions by you and a randomly drawn other player.

For each two ECUs contributed by you and the other player, the experimenter will add one ECU.

Finally, all ECUs will be shared equally between you and the other player.

[Text on bottom right-hand side]

I will contribute to common treasury [X]

6w

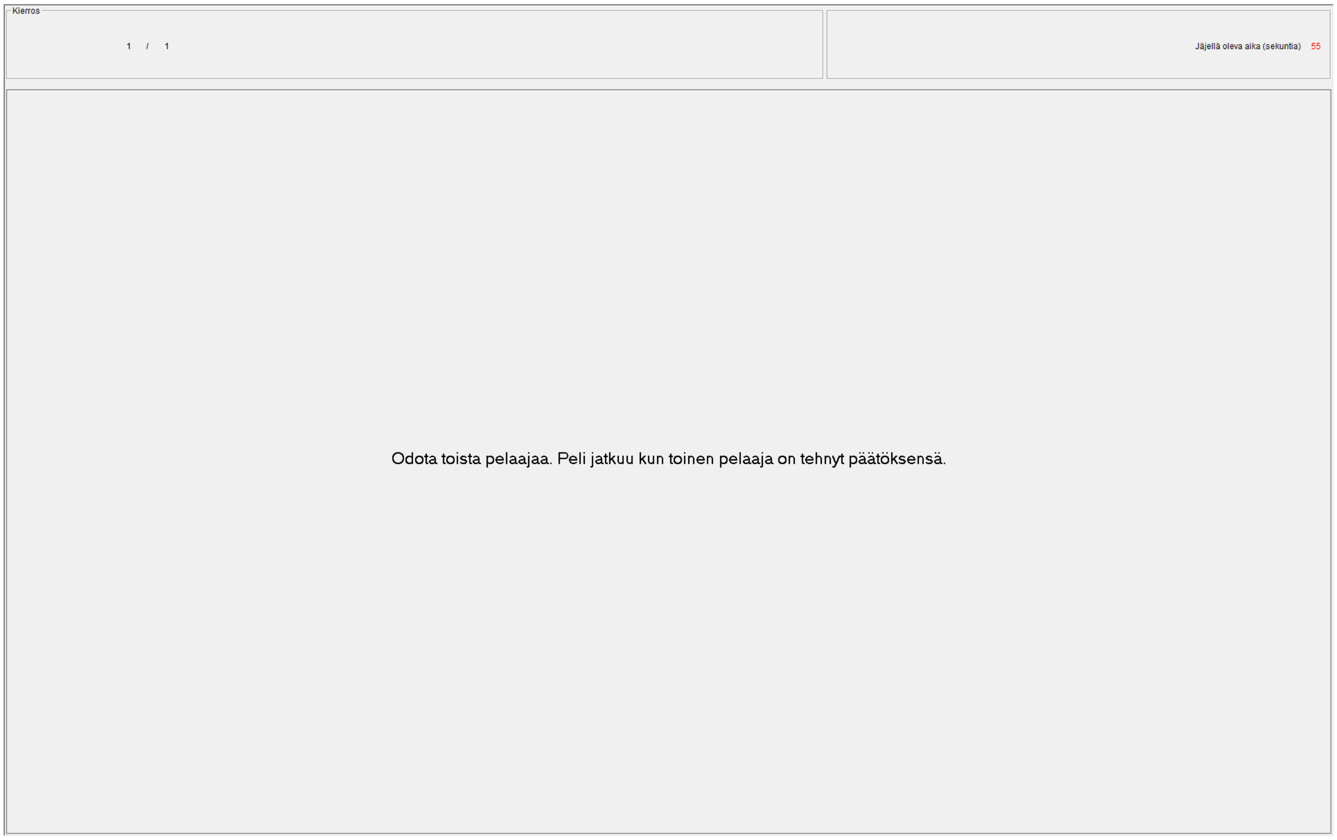

Wait for the other player. The game will resume after the other player has reached his/her decision.

7

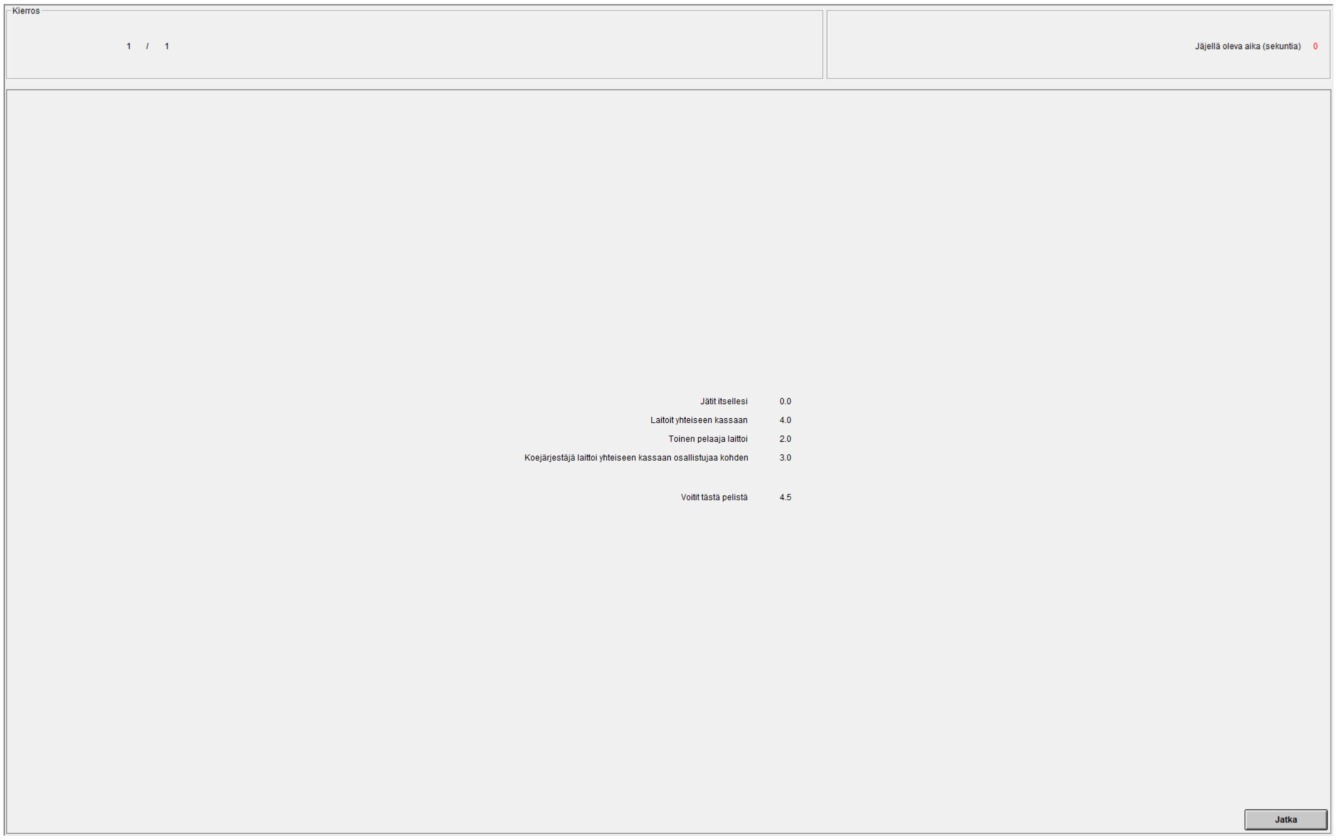

You kept to yourself	[Y]
You contributed to common treasury	[Y]
The other player contributed	[Y]
Experimenter added to common treasury per player	[Y]
Your profit from this game	[Y]

8

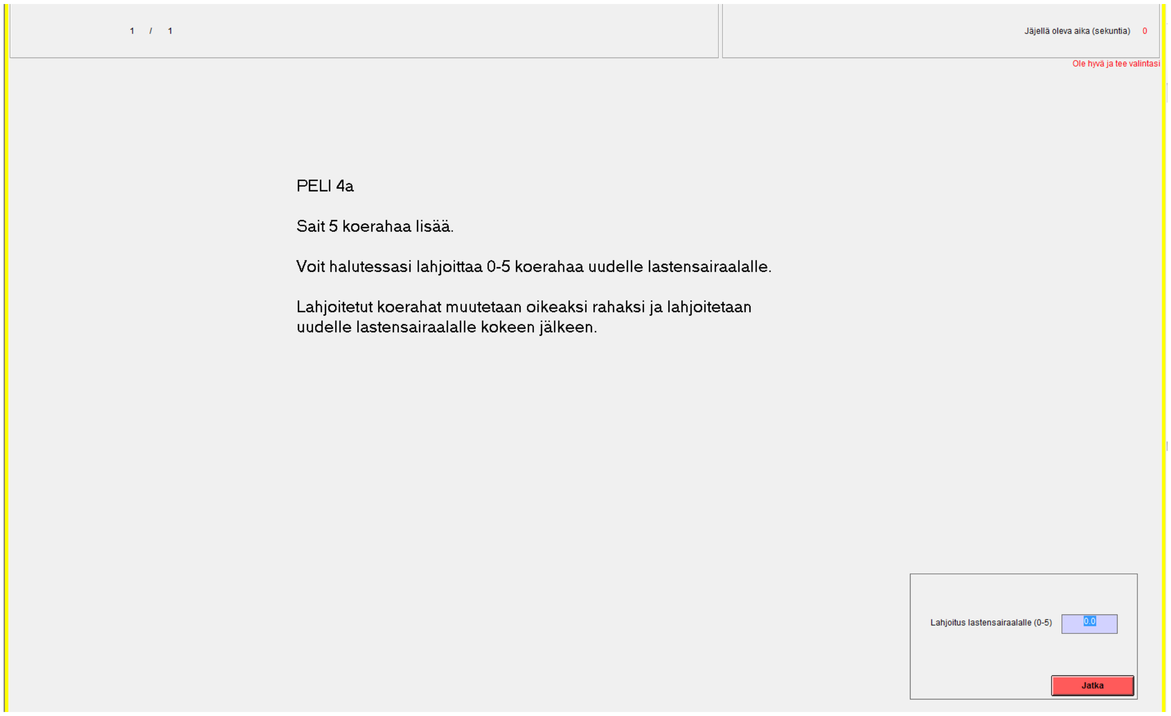

[Text at top]

GAME 4a

You have received an additional 5 ECUs.

If you want, you can donate 0-5 ECUs to the New Children’s Hospital Project 2017.

Donated ECUs will be exchanged for real money and donated to the New Children’s Hospital after the experiment.

[Text on bottom right-hand side]

Donation to the New Children’s Hospital (0-5) [X]

8w

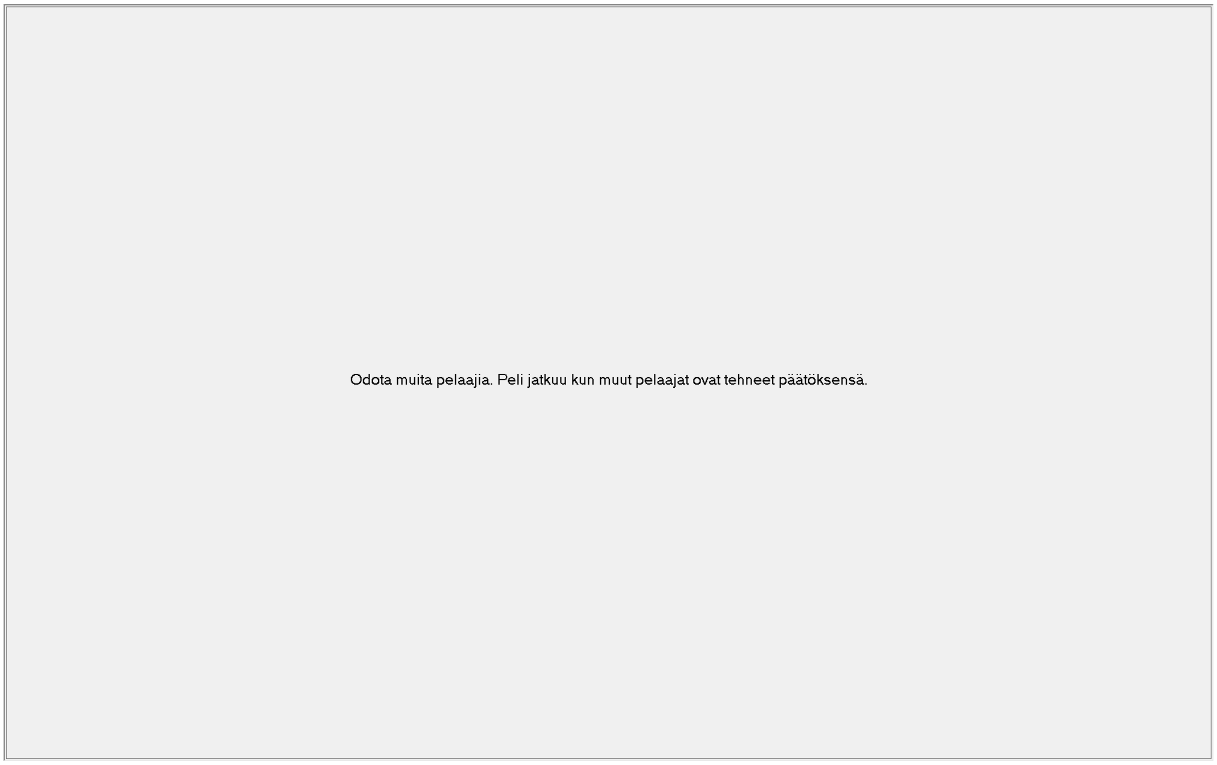

Wait for the other players. The game will resume after the other players have reached their decisions.

9

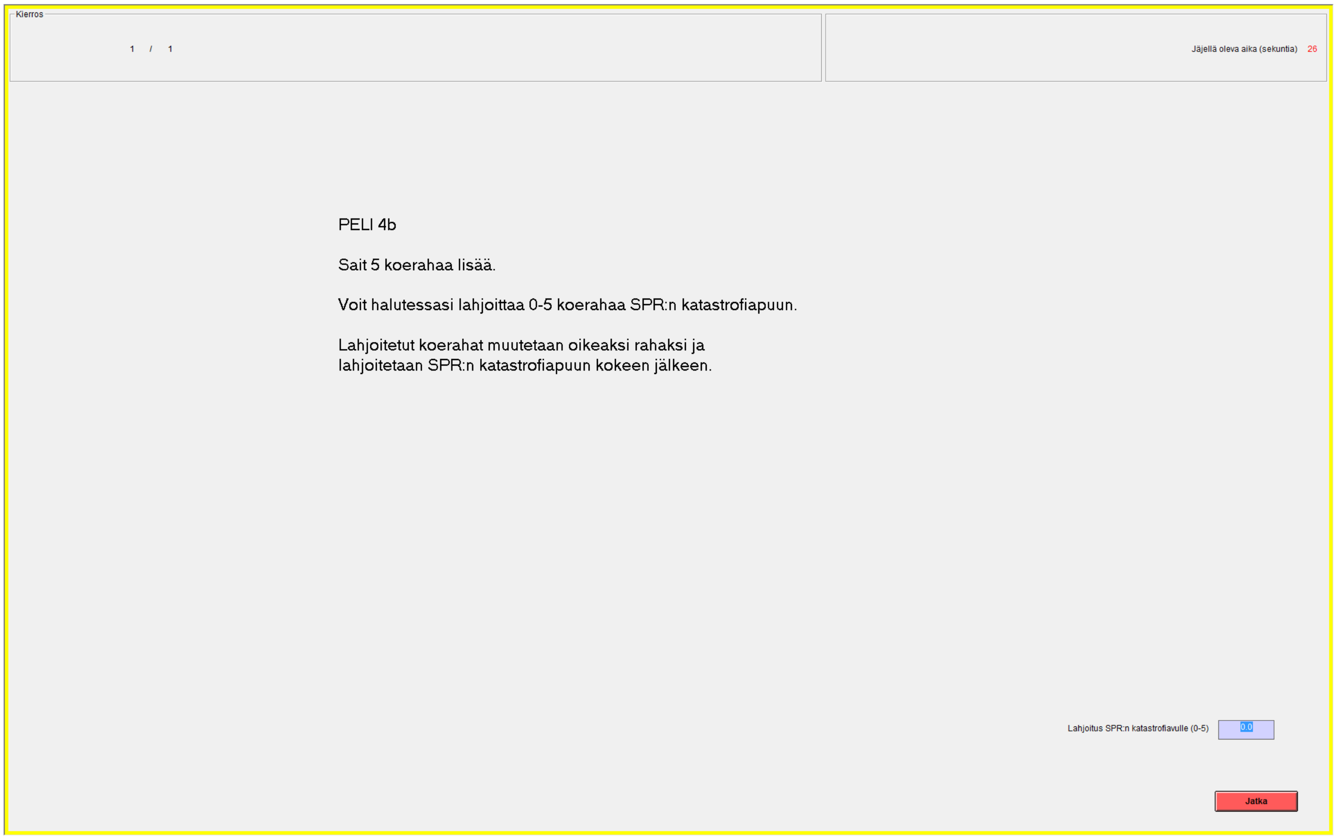

GAME 4b

You have received an additional 5 ECUs.

If you want, you can donate 0-5 ECUs to the Finnish Red Cross Disaster Relief Fund.

Donated ECUs will be exchanged for real money and donated to the Finnish Red Cross Disaster Relief Fund after the experiment.

[Text on bottom right-hand side]

Donation to the Finnish Red Cross Disaster Relief Fund (0-5) [X]

10

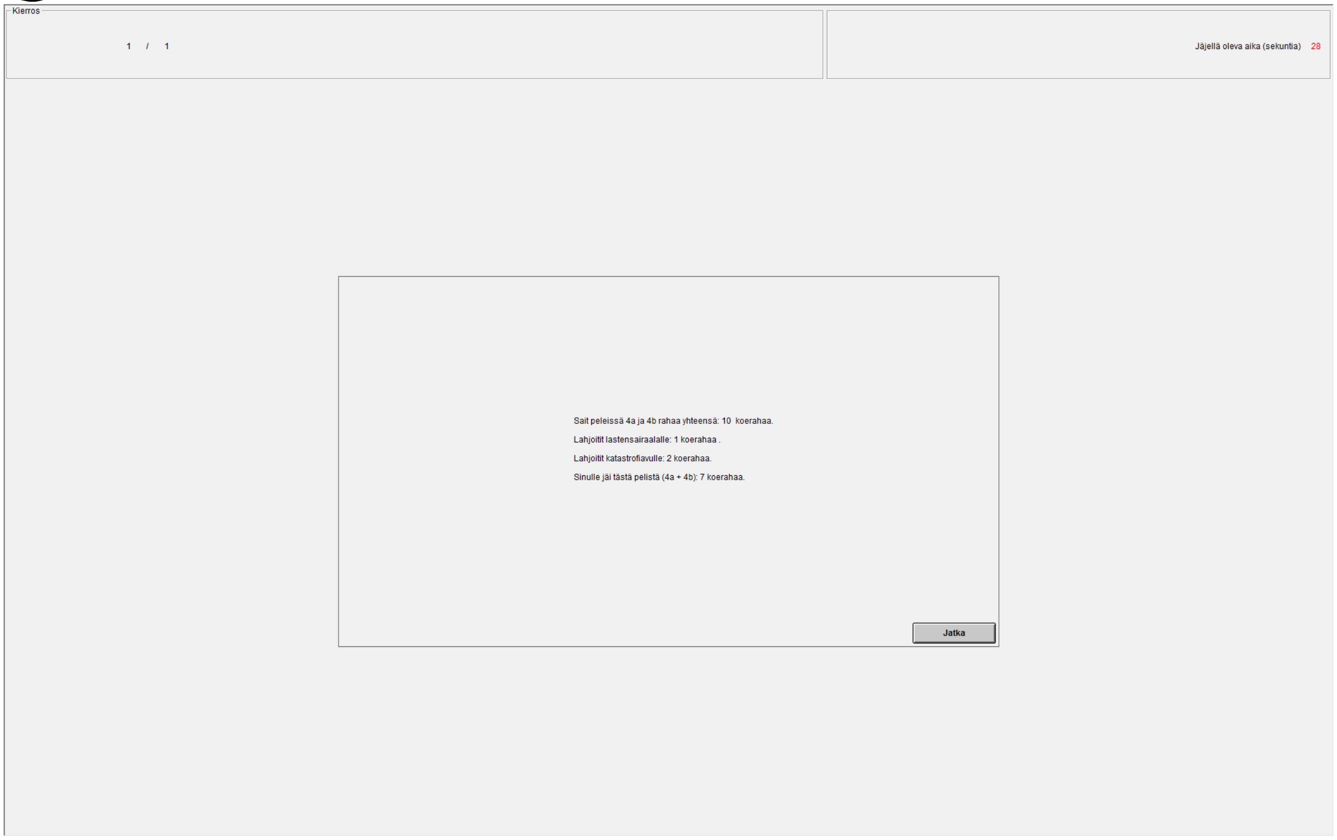

In games 4a and 4b, you received a total of: 10 ECUs

You donated to the New Children’s Hospital Project: [Y] ECUs

You donated to the Disaster Relief Fund: [Y] ECUs

From this game, you were left with (4a + 4b): [Y] ECUs

11

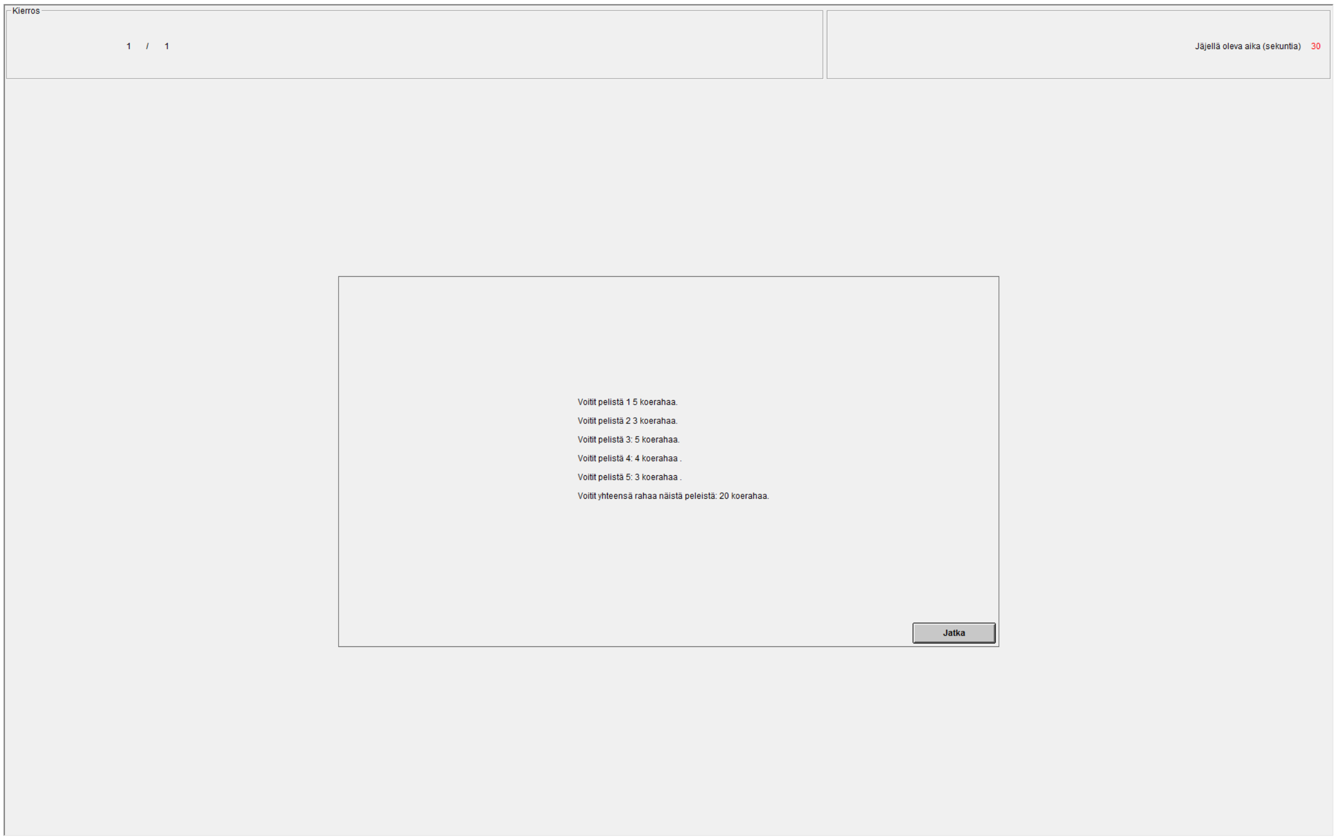

You won from game 1: [Y] ECUs

You won from game 2: [Y] ECUs

You won from game 3: [Y] ECUs

You won from game 4: [Y] ECUs

You won from game 5: [Y] ECUs

You won in total from these games: [Y] ECUs

11w

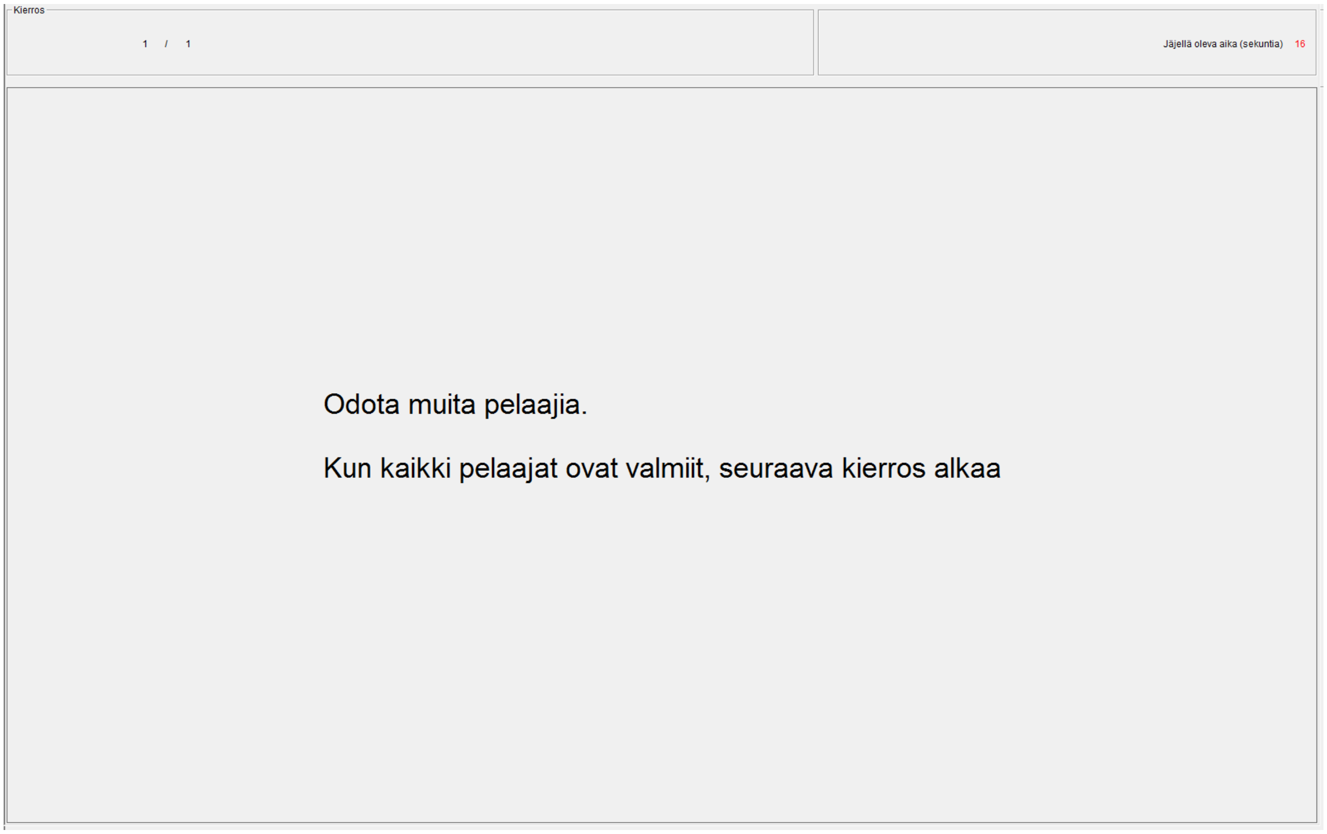

Wait for the other players.

When all the players are ready, the following round will begin.

12

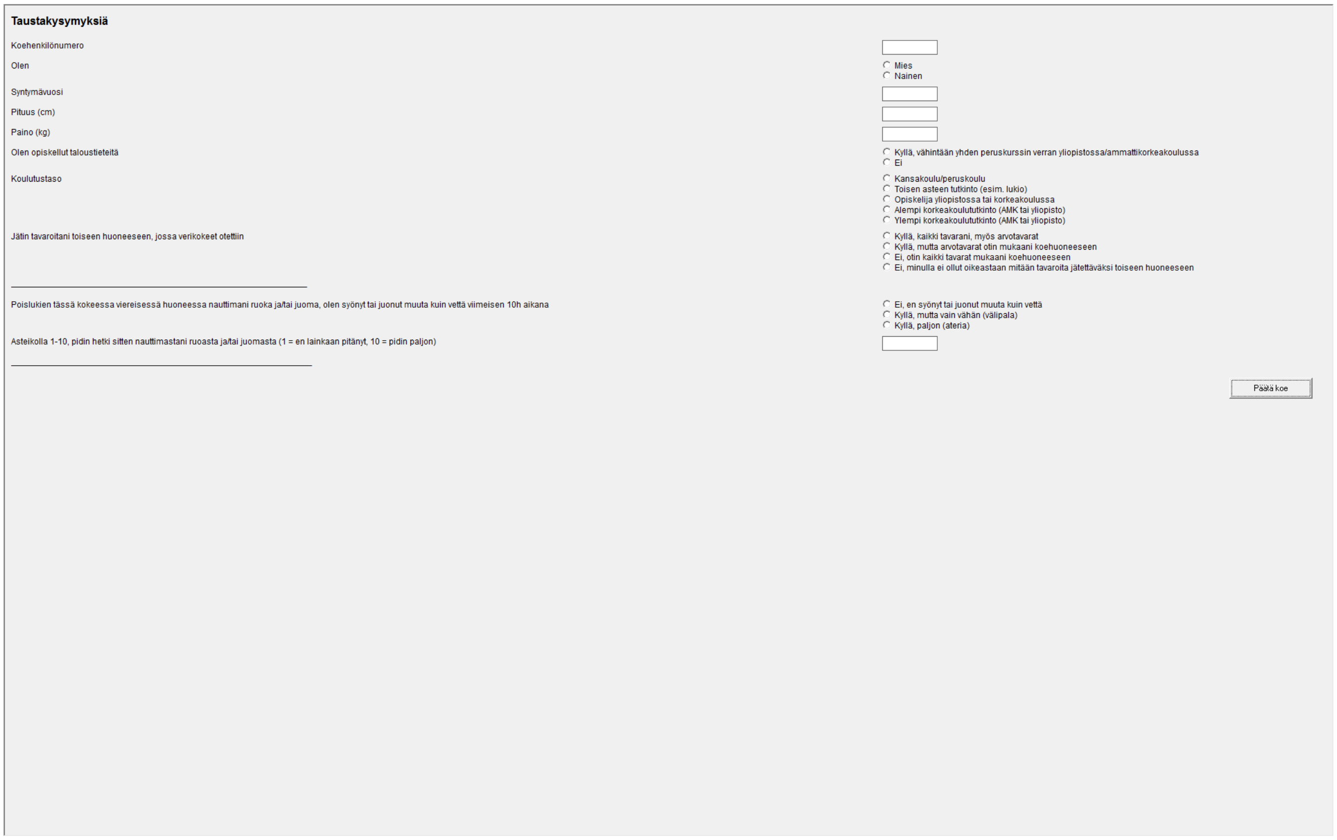

[Questions and choices for answer with [X] indicating an open field and "/" a menu of choices]

Background questions

Participant number	[X]
I am	[Male/Female]
Birth year	[X]
Height (cm)	[X]
Weight (kg)	[X]
I have studied economics	[Yes, at least one basic course at college level / No]
Level of education	[Primary school / Secondary degree including high school / Student at college or higher / B.Sc. degree / M.Sc. or higher degree]

At this experiment, I left my belongings in the other room where blood samples were taken

[Yes, all my belongings, including valuables / Yes, but I took my valuables with me to the second laboratory room / No, I took all my belongings with me to the second laboratory room / No, I did not really have anything to leave in the other room]

Excluding food and drink offered in the other room during this experiment, I have eaten or drunk nothing other than water during the past 10 hours

[No, I have not eaten or drunk anything other than water / Yes, but only a little (snack) / Yes, a lot (meal)]

On a scale of 1-10, I liked the food or drink consumed a while ago (1 = did not like at all, 10 = liked very much) [X]

[Text on the button]

Complete experiment
